# Creatine uptake regulates CD8 T cell antitumor immunity

**DOI:** 10.1084/jem.20182044

**Published:** 2019-10-18

**Authors:** Stefano Di Biase, Xiaoya Ma, Xi Wang, Jiaji Yu, Yu-Chen Wang, Drake J. Smith, Yang Zhou, Zhe Li, Yu Jeong Kim, Nicole Clarke, Angela To, Lili Yang

**Affiliations:** 1 Department of Microbiology, Immunology & Molecular Genetics, University of California, Los Angeles, Los Angeles, CA; 2 Eli and Edythe Broad Center of Regenerative Medicine and Stem Cell Research, University of California, Los Angeles, Los Angeles, CA; 3 Jonsson Comprehensive Cancer Center, the David Geffen School of Medicine, University of California, Los Angeles, Los Angeles, CA; 4 Molecular Biology Institute, University of California, Los Angeles, Los Angeles, CA

## Abstract

T cells demand massive energy to combat cancer; however, the metabolic regulators controlling antitumor T cell immunity have just begun to be unveiled. When studying nutrient usage of tumor-infiltrating immune cells in mice, we detected a sharp increase of the expression of a *CrT* (*Slc6a8*) gene, which encodes a surface transporter controlling the uptake of creatine into a cell. Using *CrT* knockout mice, we showed that creatine uptake deficiency severely impaired antitumor T cell immunity. Supplementing creatine to WT mice significantly suppressed tumor growth in multiple mouse tumor models, and the combination of creatine supplementation with a PD-1/PD-L1 blockade treatment showed synergistic tumor suppression efficacy. We further demonstrated that creatine acts as a “molecular battery” conserving bioenergy to power T cell activities. Therefore, our results have identified creatine as an important metabolic regulator controlling antitumor T cell immunity, underscoring the potential of creatine supplementation to improve T cell–based cancer immunotherapies.

## Abstract (precis)

Di Biase et al. show for the first time that creatine acts as a “molecular battery” conserving bioenergy to power CD8 T cell activities; that creatine uptake is critical in supporting antitumor T cell immunity; and that creatine supplementation holds promise for improving cancer immunotherapy.

## Graphical Abstract

**Figure:**
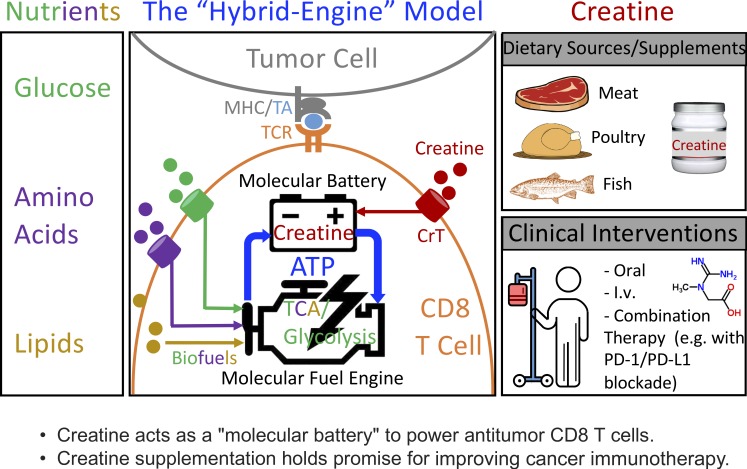

## Introduction

T cells play a central role in mediating and orchestrating immune responses against cancer; therefore, they are attractive therapeutic targets for treating cancer (Couzin-Frankel, 2013; Page et al., 2014; Ribas, 2015; Rosenberg and Restifo, 2015; Baumeister et al., 2016; Lim and June, 2017). The maintenance and activation of T cells are energy-demanding activities, requiring the use of bioenergy in the form of ATP (Fox et al., 2005). Distinct metabolic programs are used by T cells to generate ATP to support their diverse homeostatic and effector functions (Fox et al., 2005; O’Neill et al., 2016; Kidani and Bensinger, 2017; Zeng and Chi, 2017). In the tumor microenvironment, T cells face the special challenge of competing with fast-growing tumor cells for metabolic fuel such as glucose, amino acids, and lipids, which can be limiting (McCarthy et al., 2013). Therefore, an efficient and economical bioenergy metabolism is needed for tumor-infiltrating T cells to mount and sustain effective anticancer responses (Siska and Rathmell, 2015). However, the study of metabolic regulators controlling antitumor T cell immunity has just begun (Chang and Pearce, 2016; Ho and Kaech, 2017; Kishton et al., 2017; Patel and Powell, 2017). Here we show that creatine is a critical molecule buffering ATP levels in cancer-targeting CD8 T cells through maintaining a readily available high-energy phosphate reservoir (Wyss and Kaddurah-Daouk, 2000). We found that tumor-infiltrating immune cells (TIIs) up-regulated their expression of the creatine transporter gene (*Slc6a8* or *CrT*), which encodes a surface transporter controlling the uptake of creatine into a cell (Wyss and Kaddurah-Daouk, 2000). Creatine uptake deficiency severely impaired CD8 T cell responses to tumor challenge in vivo and to antigen stimulation in vitro, while supplementation of creatine through either direct administration or dietary supplement significantly suppressed tumor growth in multiple mouse tumor models. Notably, the combination of creatine supplementation with a checkpoint inhibitor blockade treatment, such as the PD-1/PD-L1 blockade, showed a synergistic tumor suppression effect, suggesting that creatine supplementation can be a valuable component for combination cancer immunotherapies. Therefore, our results have identified creatine as an important “molecular battery” that conserves bioenergy to enhance antitumor T cell immunity, underscoring the potential of creatine supplementation to improve T cell–based cancer immunotherapies.

## Results

### Creatine transporter gene (*CrT*) is up-regulated in TIIs

**Figure 1.**
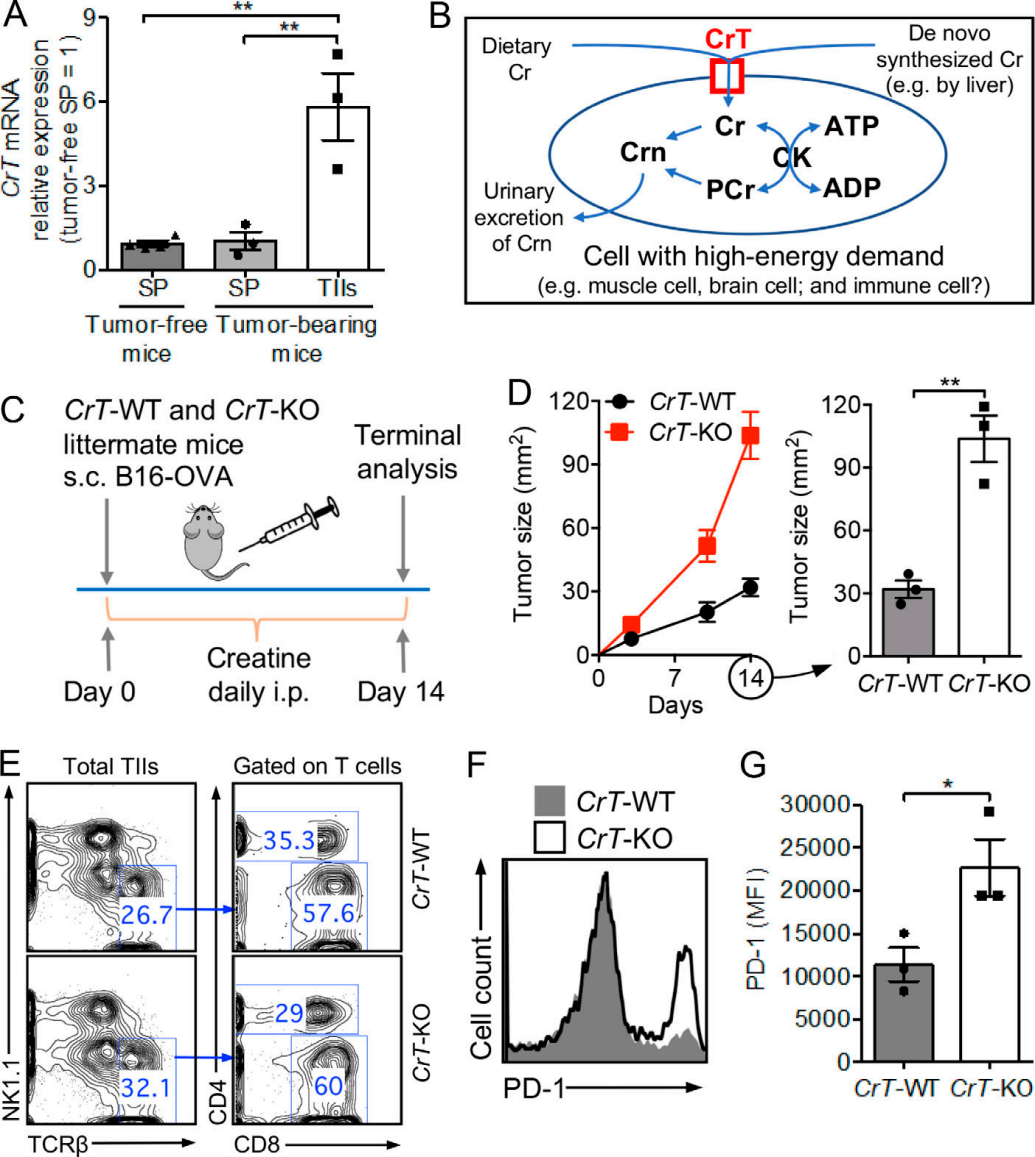
***CrT*-KO mice show impeded control of tumor growth. (A)** Creatine transporter (*CrT* or *Slc6a8*) mRNA expression in spleen (SP) cells and TIIs in a mouse B16-OVA melanoma model (*n* = 3–4) measured by qPCR. Cells were collected on day 14 after tumor challenge. **(B)** Diagram showing creatine uptake and creatine-mediated bioenergy buffering in cells with high-energy demand. Cr, creatine; PCr, phospho-creatine; Crn, creatinine; CK, creatine kinase. **(C–G)** Study of B16-OVA tumor growth in *CrT*-WT or *CrT*-KO littermate mice. **(C)** Experimental design. **(D)** Tumor growth (*n* = 3). **(E–G)** On day 14, tumors were collected from experimental mice, and TIIs were isolated for further analysis. **(E)** FACS plots showing the detection of tumor-infiltrating CD4 and CD8 T cells (gated as TCRβ^+^CD4^+^ and TCRβ^+^CD8^+^ cells, respectively). **(F)** FACS plot showing PD-1 expression on tumor-infiltrating CD8 T cells. **(G)** Quantification of F (*n* = 3). Representative of two (A) and three (C–G) experiments, respectively. Data are presented as the mean ± SEM. *, P < 0.05; **, P < 0.01 by one-way ANOVA (A) or Student’s *t* test (D and G). See also Fig. S1.

To identify metabolic regulators controlling tumor-fighting immune cells, we grew solid B16-OVA melanoma tumors in C57BL/6J mice, isolated TIIs, and then studied their gene expression profile relevant to nutrient usage using quantitative RT-PCR (qPCR). Immune cells isolated from the spleen of tumor-bearing or tumor-free mice were included as controls. Interestingly, in addition to the change of genes involved in the classic glucose/lipid/amino acid metabolic pathways (Fox et al., 2005), we detected a sharp increase of the expression of a *CrT* (*Slc6a8*) gene in TIIs (Fig. 1 A). *CrT* is an X-linked gene encoding a surface transporter (creatine transporter [CrT]) that controls the uptake of creatine into a cell in an Na^+^/K^+^-dependent manner, where creatine is used to store high-energy phosphates and to buffer intracellular ATP levels through a CK/PCr/Cr (creatine kinase/phospho-creatine/creatine) system (Fig. 1 B; Wyss and Kaddurah-Daouk, 2000).

Creatine is a nitrogenous organic acid that naturally occurs in vertebrates. It is mainly produced in the liver and kidneys but predominantly stored in skeletal muscle (Wyss and Kaddurah-Daouk, 2000). For humans, diet is also a major source of creatine (Wyss and Kaddurah-Daouk, 2000). Expression of CrT is important for cells demanding high energy, such as muscle cells and brain cells; in humans, CrT deficiency has been associated with muscle diseases and neurological disorders (Wyss and Kaddurah-Daouk, 2000). On the other hand, oral creatine supplements have been broadly used by bodybuilders and athletes to gain muscle mass and to improve performance (Kreider et al., 2017). However, the function of CrT/creatine outside of the muscle and brain tissues is largely unknown. Since we found up-regulated *CrT* gene expression in TIIs, we asked if the CrT/creatine system might also regulate the energy metabolism of tumor-fighting immune cells, in particular CD8 cytotoxic T cells, which have a massive demand for energy and can benefit from an energy storage/ATP buffering system (Fig. 1 B).

### *CrT*-KO mice show impeded control of tumor growth

To address this question, we began by studying *CrT*-KO mice (Fig. S1 A; Skelton et al., 2011). Despite their smaller body size, *CrT*-KO mice contained normal numbers of immune cells, including T cells, proportional to their body weight (Fig. S1, B–G). Before tumor challenge, these T cells displayed a typical naive phenotype (CD25^lo^CD69^lo^CD62L^hi^CD44^lo^; Fig. S1 H). In a B16-OVA melanoma model, tumor growth was accelerated in *CrT*-KO mice compared with their *CrT*-WT littermates (Fig. 1, C and D). In *CrT*-KO mice, tumor-infiltrating CD8 T cells expressed higher levels of PD-1, which has been associated with bioenergy insufficiency and T cell exhaustion, indicating that CrT deficiency may impact antitumor T cell activities (Fig. 1, E–G; Chang et al., 2015; Wherry and Kurachi, 2015; Bengsch et al., 2016). Of note, the regular mouse diet (PicoLab Rodent Diet 20) does not contain creatine; therefore, to mimic the supply of creatine from dietary resources in humans, we supplied creatine to experimental mice via i.p. injection (Fig. 1 C). Without i.p. injection of creatine, no B16-OVA tumor growth difference was observed between *CrT*-WT and *CrT*-KO mice, likely due to the lack of sufficient creatine supply in these experimental mice to read out the creatine uptake difference between *CrT*-WT and *CrT*-KO mice (Fig. S1, I and J). Interestingly, study of *CrT* gene expression in tumor-infiltrating WT CD8 T cell subsets showed an up-regulation of *CrT* gene expression that was more significant in the PD-1^hi^ subset than in the PD-1^lo^ subset, suggesting a possible feedback loop in PD-1^hi^ CD8 T cells that compensates for bioenergy insufficiency by increasing creatine uptake (Fig. S1 K). In particular, the PD-1^hi^Tim-3^hi^LAG-3^hi^ tumor-infiltrating CD8 T cells, which are considered to be the most “exhausted,” expressed the highest levels of *CrT*, suggesting that these cells may also benefit the most from creatine supplementation treatment (Fig. S1 K; Nguyen and Ohashi, 2015; Wherry and Kurachi, 2015).

### Creatine uptake deficiency directly impairs antitumor T cell immunity

**Figure 2.**
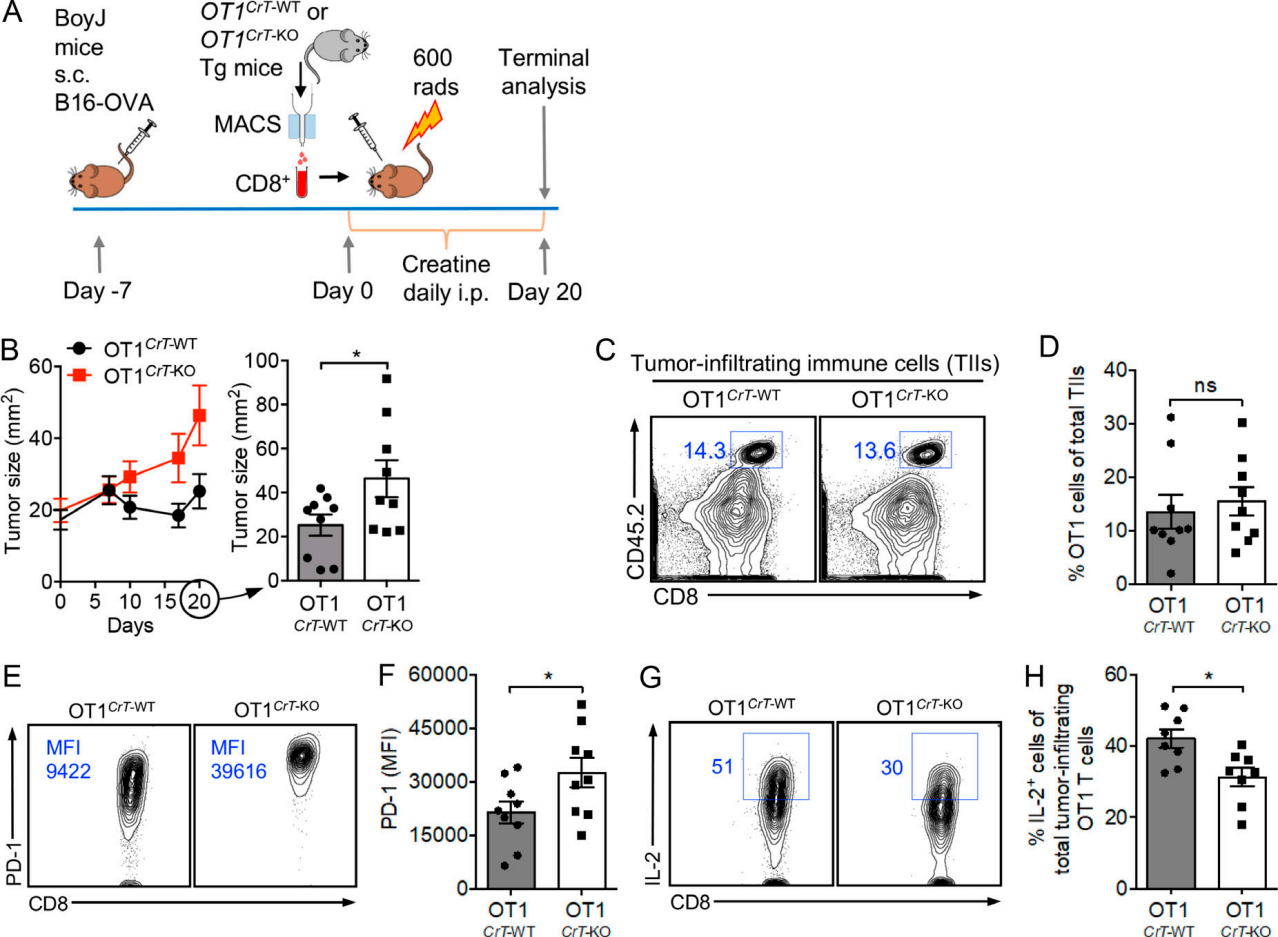
**Creatine uptake deficiency directly impairs antitumor T cell immunity.** B16-OVA tumor growth in BoyJ mice was studied. BoyJ mice received adoptive transfer of OVA-specific OT1 Tg CD8 T cells that were either WT or KO of *CrT* gene (OT1*^CrT^*^-WT^ or OT1*^CrT^*^-KO^ cells, respectively). **(A)** Experimental design. **(B)** Tumor growth (*n* = 9). **(C–H)** On day 20, tumors were collected from experimental mice, and TIIs were isolated for further analysis. **(C)** FACS plots showing the detection of tumor-infiltrating OT1 T cells (gated as CD45.2^+^CD8^+^ cells). **(D)** Quantification of C (*n* = 9). **(E)** FACS plots showing PD-1 expression on tumor-infiltrating OT1 T cells. **(F)** Quantification of E (*n* = 9). **(G)** FACS plots showing intracellular IL-2 production of tumor-infiltrating OT1 T cells. Before intracellular cytokine staining, TIIs were stimulated with PMA and ionomycin in the presence of GolgiStop for 4 h. **(H)** Quantification of G (*n* = 8). Representative of two experiments (A–H). Data are presented as the mean ± SEM. ns, not significant; *, P < 0.05 by Student’s *t* test. See also Fig. S2.

To study the direct regulation of immune cells by CrT, we reconstituted WT BoyJ mice with bone marrow (BM) cells from either *CrT*-WT or *CrT*-KO donor mice and then challenged recipient mice with B16-OVA tumor cells (Fig. S2 A). CrT deficiency did not impair the reconstitution of an immune system in the recipient mice (Fig. S2, B and C), but it did impede the capacity of the reconstituted immune system to control tumor growth (Fig. S2 D). To further study the direct regulation of tumor-specific CD8 T cells by CrT, we bred *CrT*-KO mice with *OT1* transgenic (Tg) mice and generated *OT1*Tg*^CrT^*^-KO^ mice producing OVA-specific CD8 T cells deficient in CrT (Fig. S2 E). We isolated OT1*^CrT^*^-WT^ and OT1*^CrT^*^-KO^ CD8 T cells (Fig. S2 F) and separately transferred these T cells into BoyJ WT mice bearing preestablished B16-OVA tumors (Fig. 2 A). Compared with OT1*^CrT^*^-WT^ cells, OT1*^CrT^*^-KO^ cells were less effective in controlling tumor growth (Fig. 2 B). Although OT1*^CrT^*^-KO^ cells infiltrated tumors and showed an antigen-experienced phenotype (CD62L^lo^CD44^hi^; Fig. 2, C and D; and Fig. S2 G), these T cells expressed higher levels of PD-1 (Fig. 2, E and F; and Fig. S2 G) and produced a smaller amount of effector cytokines, including IL-2 (Fig. 2, G and H) and IFN-γ (Fig. S2, H and I), compared with OT1*^CrT^*^-WT^ cells. Similarly, mice in these tumor experiments received i.p. injection of creatine to compensate for the lack of creatine supply from mouse diet (Fig. 1 C, Fig. S2 A, and Fig. 2 A). Collectively, these in vivo data demonstrate that creatine uptake deficiency directly impairs antitumor immunity, especially the antitumor efficacy of tumor antigen–specific CD8 cytotoxic T cells.

### Creatine uptake regulates CD8 T cell response to antigen stimulation

**Figure 3.**
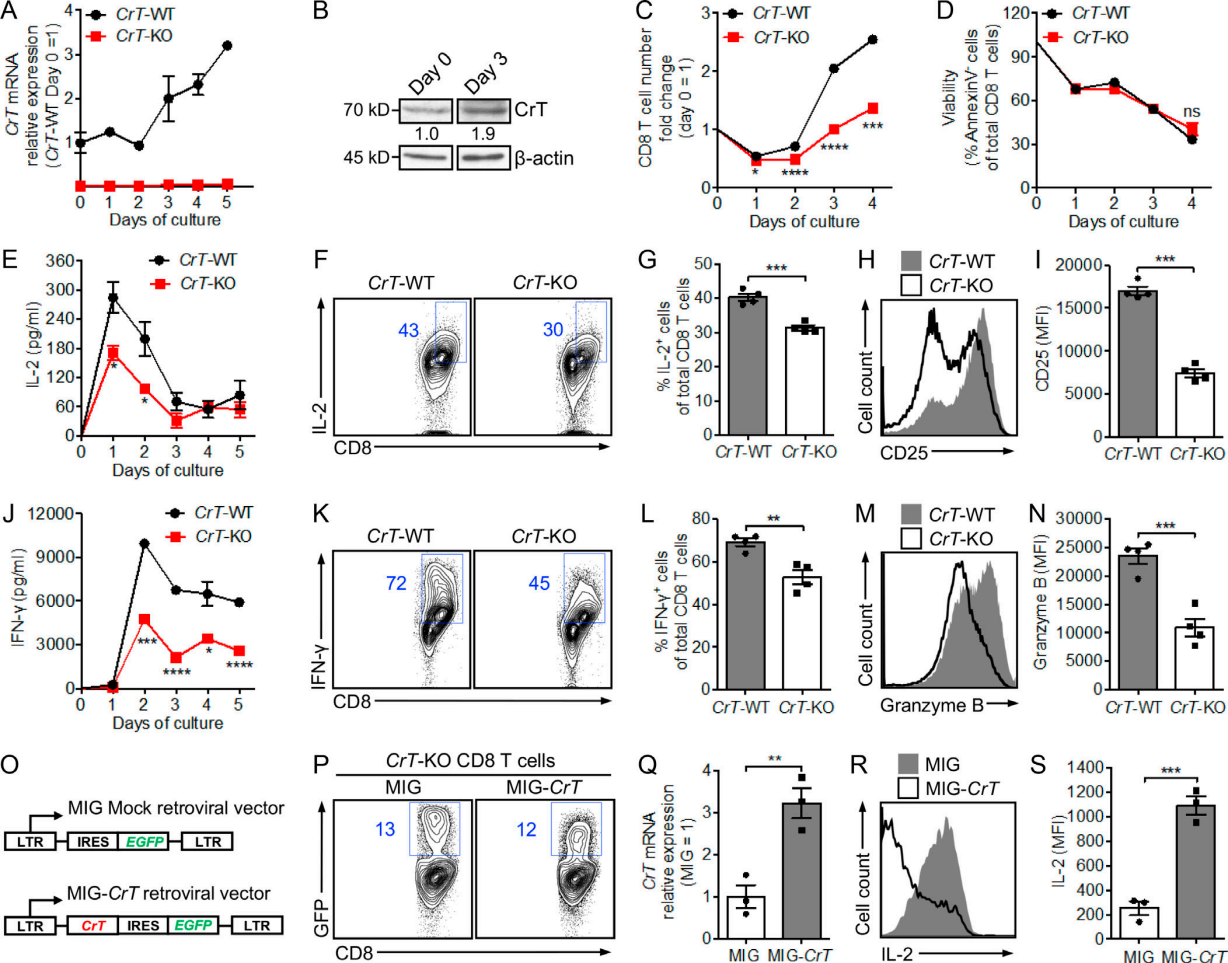
**Creatine uptake regulates CD8 T cell response to antigen stimulation. (A–N)** CD8 T cells were purified from *CrT*-WT or *CrT*-KO mice and stimulated in vitro with plate-bound anti-CD3 (5 µg/ml; *n* = 3–4). The analyses of *CrT* mRNA expression (A), CrT protein expression (B), cell proliferation (C), cell survival (D), effector cytokine production (E–G and J–L), activation marker expression (H and I), and cytotoxic molecule production (M and N) are shown, either over a 4- to 5-d time course (A, C, D, E, and J) or 48 h after anti-CD3 stimulation (F–I and K–N). **(O–S)** *CrT*-KO CD8 T cells were stimulated in vitro with anti-CD3 and transduced with a MIG-*CrT* retrovector (O; *n* = 3). The analyses of retrovector transduction rate (P), *CrT* mRNA expression (Q), and IL-2 effector cytokine production (R and S) 96 h after stimulation are shown. Representative of two (O–S) and three (A–N) experiments, respectively. Data are presented as the mean ± SEM. ns, not significant; *, P < 0.05; **, P < 0.01; ***, P < 0.001; ****, P < 0.0001 by Student’s *t* test. See also Fig. S3.

Next, to study how creatine uptake regulates CD8 T cell response to antigen stimulation, we isolated CD8 T cells from *CrT*-WT or *CrT*-KO littermate mice, followed by stimulating these cells in vitro with anti-CD3. A standard T cell culture medium was used, which comprised 10% FBS as the source of creatine (Fig. S3 A). After stimulation, WT CD8 T cells showed up-regulated expression of *CrT* mRNA (Fig. 3 A) and CrT protein (Fig. 3 B), indicating the induction of CrT expression by TCR signaling and suggesting, in turn, the need for activated CD8 T cells to uptake more creatine. Compared with their *CrT*-WT counterparts, *CrT*-KO CD8 T cells showed a reduction in almost all aspects of T cell activation, including cell proliferation (Fig. 3 C), effector cytokine production (e.g., IL-2 and IFN-γ; Fig. 3, E–G and J–L), surface activation marker expression (e.g., CD25; Fig. 3, H and I), and cytotoxic molecule production (e.g., Granzyme B; Fig. 3, M and N). Cell survival, studied via Annexin V and 7-aminoactinomycin D (7-AAD) staining, was not affected over a 4-d cell culture period (Fig. 3 D). Study of OVA-specific OT1*^CrT^*^-KO^ CD8 T cells gave similar results (Fig. S3, B–J), suggesting a general role of CrT in regulating CD8 T cells of diverse antigen specificities. To verify whether creatine uptake deficiency directly contributed to the hyporesponsiveness of the *CrT*-KO CD8 T cells, we conducted a rescue experiment. We constructed a MIG-*CrT* retroviral vector (Fig. 3 O), used this vector to transduce *CrT*-KO CD8 T cells, and finally achieved overexpression of *CrT* in these cells (Fig. 3, P and Q). CrT overexpression rescued the activation of *CrT*-KO CD8 T cells and improved their production of multiple effector cytokines (Fig. 3, R and S; and Fig. S3, K and L). Taken together, these data indicate that CD8 T cells, after antigen stimulation, increase their capacity to uptake creatine that is critical for them to manifest a productive effector T cell response.

### Creatine uptake modulates CD8 T cell activation by regulating T cell ATP/energy buffering

**Figure 4.**
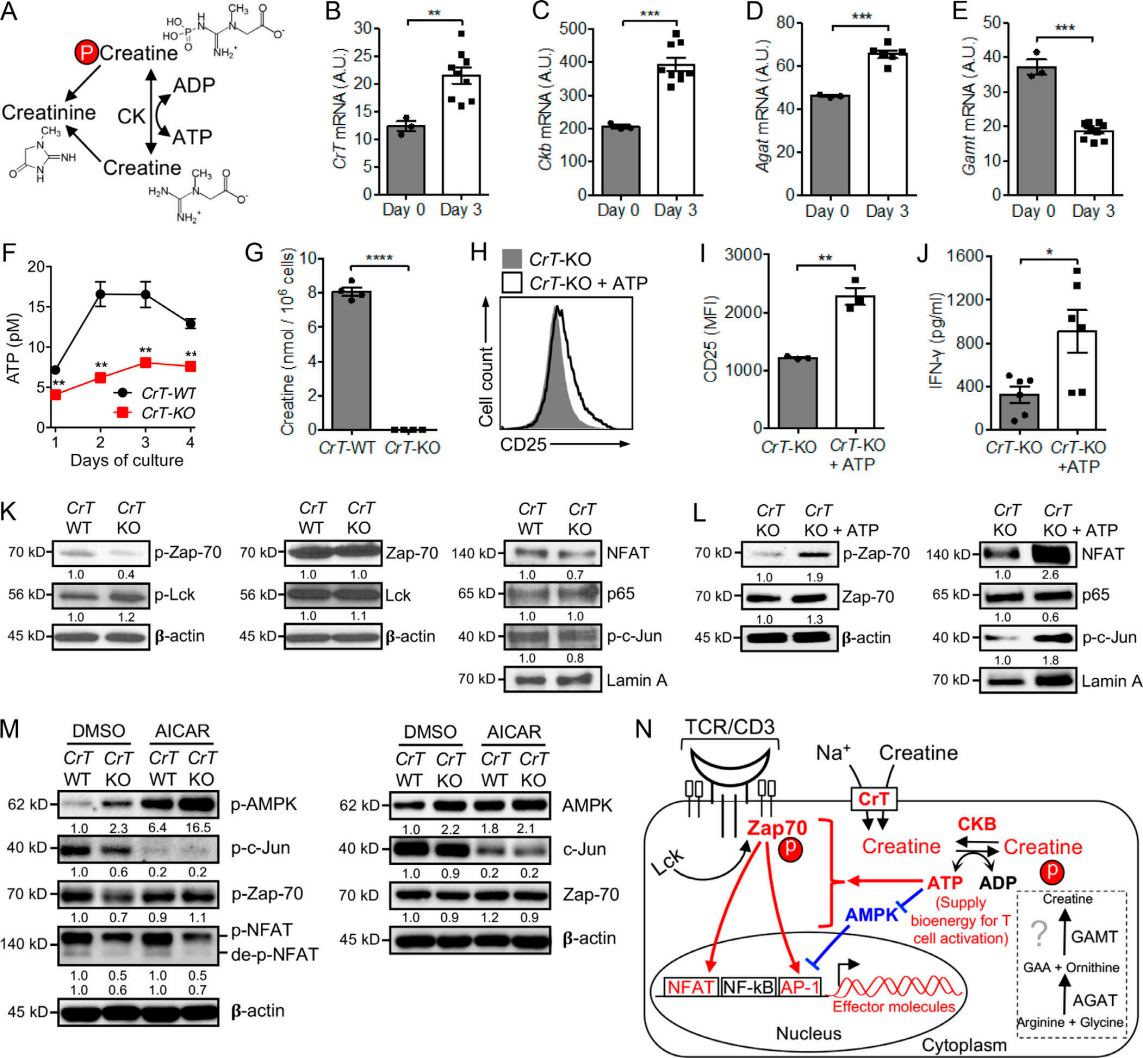
**Creatine uptake modulates CD8 T cell activation by regulating T cell ATP/energy buffering. (A)** Schematic of creatine-mediated ATP/energy buffering. **(B–E)** *CrT*-WT CD8 T cells were stimulated with anti-CD3 and analyzed for mRNA expression of creatine transporter (*CrT*; B), *Ckb* (C), and two enzymes controlling the de novo synthesis of creatine, *Agat* (D) and *Gamt* (E). *n* = 3–9. A.U., artificial unit relative to *Ube2d2*. **(F and G)** *CrT*-WT and *CrT*-KO CD8 T cells were stimulated with anti-CD3 and analyzed for intracellular levels of ATP over time (F) and creatine at 48 h (G). *n* = 4. **(H–J)** *CrT*-KO CD8 T cells were stimulated with anti-CD3, with or without ATP supplementation (100 µm) in the culture medium, and analyzed for surface CD25 activation marker expression (H and I) and IFN-γ effector cytokine production (J) at day 3. *n* = 3–6. **(K)** Western blot analysis of TCR signaling events in *CrT*-WT and *CrT*-KO CD8 T cells. *CrT*-WT and *CrT*-KO CD8 T cells were stimulated with anti-CD3 for 48 h, rested at 4°C for 2 h, then restimulated with anti-CD3 for 30 min followed by Western blot analysis. **(L)** Western blot analysis of TCR signaling events in *CrT*-KO CD8 T cells with or without ATP supplementation. *CrT*-KO CD8 T cells were stimulated with anti-CD3 for 48 h, rested at 4°C for 2 h, then restimulated with anti-CD3 for 30 min in the presence or absence of ATP supplementation (100 µm) followed by Western blot analysis. **(M)** Western blot analysis of TCR signaling events in *CrT*-WT and *CrT*-KO CD8 T cells with or without AICAR treatment. *CrT*-WT and *CrT*-KO CD8 T cells were pretreated with AICAR (2 mM) for 30 min, then stimulated with anti-CD3 for 20 min followed by Western blot analysis. DMSO, solvent used to dissolve AICAR. **(N)** Schematic model showing creatine uptake regulation of T cell activation signaling events. The demonstrated pathways are highlighted in red and blue. Representative of two experiments (B–M). Data are presented as the mean ± SEM. *, P < 0.05; **, P < 0.01; ***, P < 0.001; ****, P < 0.0001 by Student’s *t* test. See also Fig. S4.

It has been well characterized that muscle cells and brain cells uptake creatine through CrT and then use creatine to buffer intracellular ATP levels and power cellular activities via a CK/PCr/Cr system (Wyss and Kaddurah-Daouk, 2000). Therefore, we investigated whether CD8 T cells might use a similar molecular mechanism (Fig. 4 A). After TCR stimulation, WT CD8 T cells up-regulated *CrT* gene expression, enabling the activated T cells to more effectively uptake creatine (Fig. 4 B). CD8 T cells expressed high basal levels of *Ckb* (creatine kinase brain form) gene, the expression of which was further up-regulated after TCR stimulation, maximizing the capacity of activated CD8 T cells to use the CK/PCr/Cr ATP buffering system (Fig. 4 C).

De novo synthesized creatine might be another source to feed the CK/PCr/Cr system. Consequently, we examined the expression of genes encoding the two enzymes controlling creatine biosynthesis, *Agat* (l-arginine:glycine amidinotransferase) and *Gamt* (guanidinoacetate *N*-methyltransferase). We found that CD8 T cells expressed low levels of both genes and further down-regulated the expression of *Gamt* gene after TCR stimulation (Fig. 4, D and E). Therefore, activated CD8 T cells may have limited capacity to synthesize creatine de novo and may, therefore, heavily rely on importing creatine via CrT from extracellular sources to feed the CK/PCr/Cr ATP-buffering system. In agreement with this notion, compared with *CrT*-WT CD8 T cells, activated *CrT*-KO CD8 T cells contained undetectable levels of intracellular creatine (Fig. 4 G) and significantly reduced ATP (Fig. 4 F; Wyss and Kaddurah-Daouk, 2000). The hypoactivation of *CrT*-KO CD8 T cells was rescued by supplementing ATP in T cell culture, evidenced by increased expression of T cell surface activation marker CD25 and enhanced production of effector cytokine IFN-γ (Fig. 4, H–J). Supplementing ATP further enhanced the activation of *CrT*-WT CD8 T cells (Fig. S4, A and B). ATP supplies bioenergy and phosphate group for TCR signaling events (Patel and Powell, 2017). By comparing the major TCR signaling pathways in *CrT*-WT and *CrT*-KO CD8 T cells, we found that creatine uptake deficiency impeded activation of the TCR proximal signaling molecule Zap-70 (zeta chain of TCR-associated protein kinase 70) and the downstream transcription factors NFAT and c-Jun (Jun proto-oncogene, AP-1 transcription factor subunit), which, at least partially, accounted for the hypoactivation of *CrT*-KO CD8 T cells (Fig. 4 K). Creatine supplementation significantly increased Zap-70 phosphorylation in *CrT*-WT CD8 T cells but not in *CrT*-KO CD8 T cells (Fig. S4 E). The TCR signaling deficiencies in *CrT*-KO CD8 T cells were effectively rescued by supplementing ATP to the T cell culture (Fig. 4 L).

Interestingly, compared with the activation of NFAT and AP-1, the activation of NF-κB, in particular its p65 subunit, was less sensitive to CrT deficiency–induced ATP shortage, suggesting that the NF-κB signaling pathway may better resist ATP fluctuation during T cell response (Fig. 4, K and L). AMPK (5′ adenosine monophosphate-activated protein kinase) is an enzyme that detects shifts in the AMP:ATP ratio within a cell. It serves as a nutrient and energy sensor to maintain cell energy homeostasis and has been indicated to regulate T cell metabolism and function (Tamás et al., 2006; Hardie et al., 2012; Rao et al., 2016; Ma et al., 2017). We therefore examined the possible role of AMPK in mediating the *CrT*-KO CD8 T cell hypoactivation phenotype. In correspondence with the decreased ATP levels in *CrT*-KO CD8 T cells, we detected increased activation of AMPK in these cells compared with that in *CrT*-WT CD8 T cells (Fig. 4, F and M). Treating *CrT*-WT and *CrT*-KO CD8 T cells with AICAR (5-aminoimidazole-4-carboxamide 1-β-d-ribofuranoside; an AMPK activator) markedly activated AMPK in both T cells (Fig. 4 M), and was associated with a significant reduction of AP-1 transcription factor activation (c-Jun subunit; Fig. 4 M), cell surface activation marker expression (CD25; Fig. S4 C), and effector cytokine production (IL-2; Fig. S4 D) in both T cells. Meanwhile, the activation of Zap-70 and NFAT were not affected by AICAR treatment (Fig. 4 M). Hence, creatine uptake modulation of bioenergy homeostasis in CD8 T cells may be monitored and regulated by AMPK, at least partly through AMPK regulation of the AP-1 pathway. Collectively, these results support an intriguing working model in which activated CD8 T cells (1) employ a potent creatine-mediated ATP/energy buffering system to sustain TCR signaling and power T cell effector functions, at least partly through ATP/AMPK regulation of TCR signaling pathways; and (2) rely on importing creatine via CrT from extracellular sources (Fig. 4 N).

### Creatine supplementation for cancer immunotherapy

**Figure 5.**
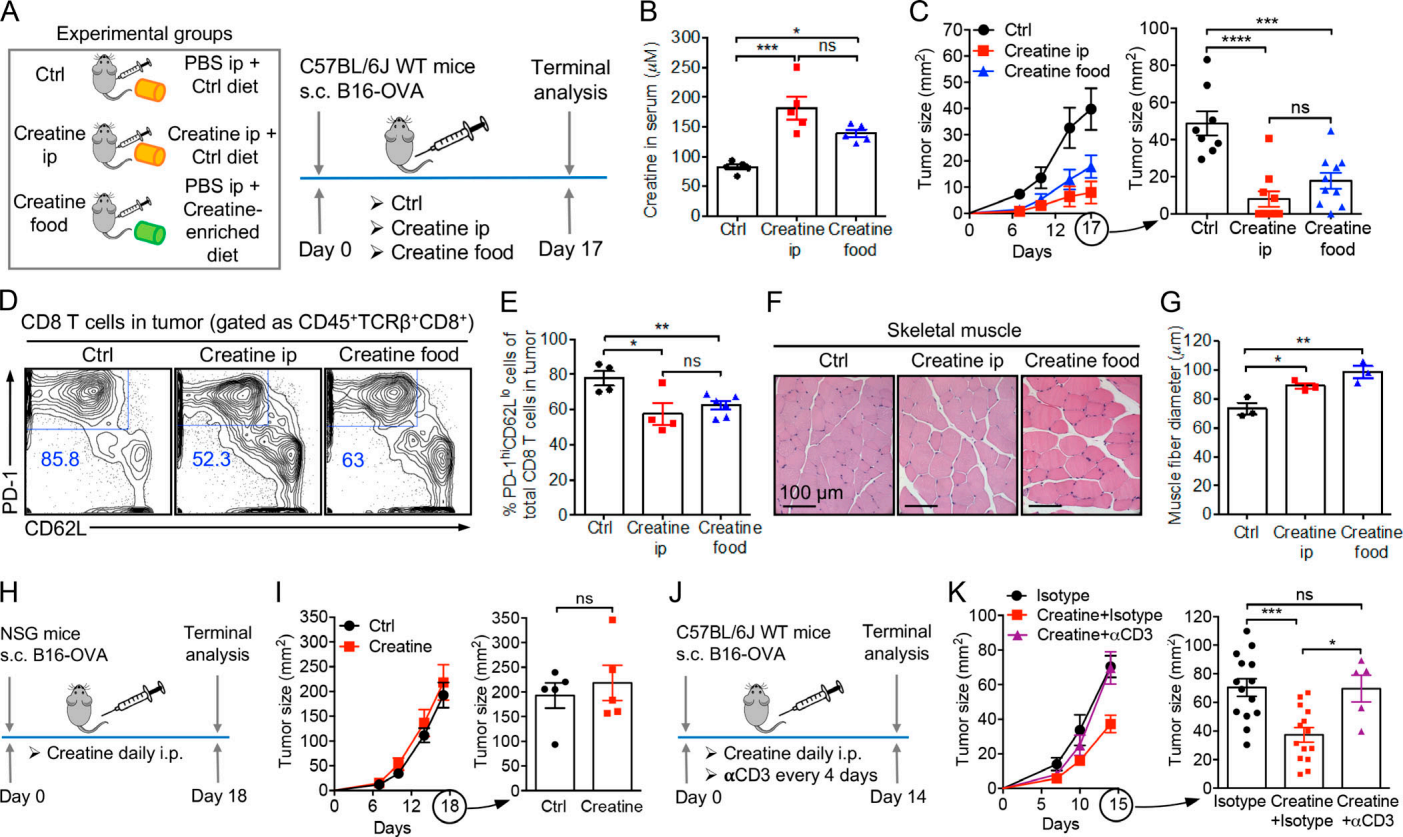
**Creatine supplementation for cancer immunotherapy. (A–G)** Studying the therapeutic potential of creatine supplementation in a B16-OVA melanoma model. **(A)** Experimental design. **(B)** Creatine levels in serum (*n* = 5). **(C)** Tumor progression (*n* = 8–10). **(D–G)** On day 17, tumors and muscles were collected from experimental mice for further analysis. **(D)** FACS plots showing the phenotype of tumor-infiltrating CD8 T cells. **(E)** Quantification of D (*n* = 4–6). **(F)** H&E-stained skeletal muscle sections. Scale bar: 100 µm. **(G)** Quantification of F (*n* = 3). **(H and I)** Studying the requirement of an intact immune system for cancer therapy effects. **(H)** Experimental design. **(I)** Tumor progression (*n* = 5). NSG, NOD/SCID/γc^−/−^ immunodeficient mice. **(J and K)** Studying the requirement of T cells for creatine cancer therapy effects. I.p. injection of an anti-CD3 depleting antibody (αCD3, clone 17A2) was used for in vivo depletion of T cells. **(J)** Experimental design. **(K)** Tumor progression (*n* = 5–9). Representative of two (H–K) and three (A–G) experiments. Data are presented as the mean ± SEM. ns, not significant; *, P < 0.05; **, P < 0.01; ***, P < 0.001; ****, P < 0.0001 by one-way ANOVA (B, C, E, G, and K) or Student’s *t* test (I). See also Fig. S5.

The “creatine-uptake/energy-buffering” working model (Fig. 4 N) opens up the possibility of reinvigorating disease-responding CD8 T cells, in particular tumor-fighting CD8 T cells, through creatine supplementation. To test this new concept of metabolic reprogramming and cancer immunotherapy, we supplemented creatine to experimental C57BL/6J WT mice in the B16-OVA melanoma model, through either i.p. injection or dietary supplementation (Fig. 5 A). Notably, the dietary supplemental dose we used (0.4 g/kg body weight) is comparable to the safe loading dose recommended for athletes (Kreider et al., 2017). Both administration routes elevated creatine concentrations in blood to a similar level (Fig. 5 B) and effectively suppressed tumor growth to a similar extent (Fig. 5 C). The tumor suppression effect was associated with a significant reduction of the “exhaustion-prone” phenotype cells (gated as PD-1^hi^CD62L^lo^) among the tumor-infiltrating CD8 T cells (Fig. 5, D and E). In agreement with the muscle-enhancement effect of creatine, we observed an enlargement of skeletal muscle fibers in mice receiving creatine supplements (Fig. 5, F and G; Wyss and Kaddurah-Daouk, 2000; Kreider et al., 2017). On the other hand, B16-OVA tumors grown in immunodeficient NSG mice (Fig. 5, H and I) or in C57BL/6J WT mice depleted of T cells via i.p. injection of an anti-CD3 depletion antibody (Fig. 5, J and K; and Fig. S5 A) could not be suppressed by creatine supplementation, confirming that the therapeutic effect of creatine supplementation is mediated by immune cells, in particular T cells. Taken together, these results demonstrate the capacity of creatine supplementation to boost antitumor T cell immunity, thus suggesting its potential as a new means of cancer immunotherapy.

### Creatine supplementation for combination cancer therapy

**Figure 6.**
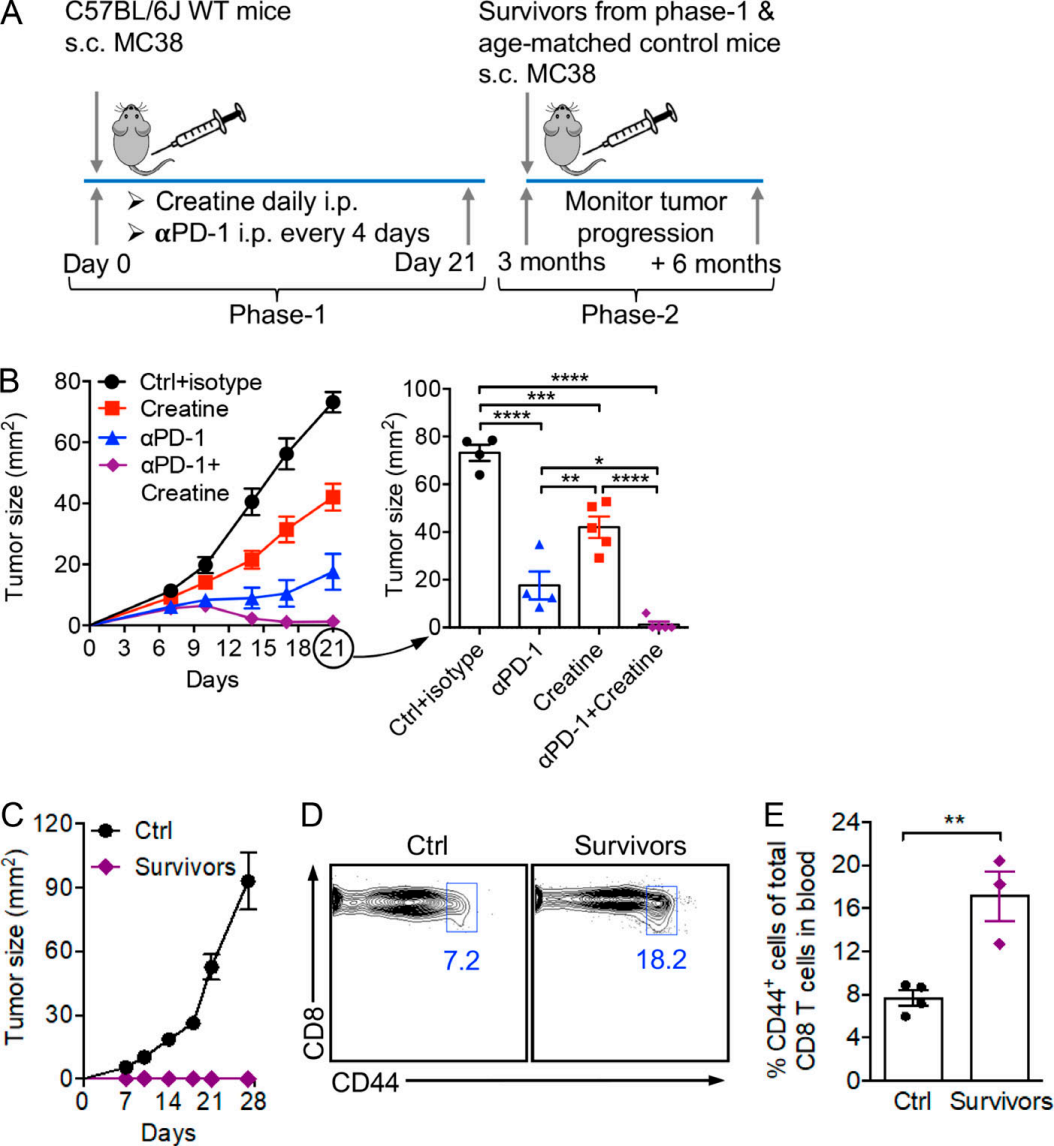
**Creatine supplementation for combination cancer therapy.** Studying the therapeutic potential of creatine supplementation in combination with anti–PD-1 (αPD-1) treatment in an MC38 colon cancer model. **(A)** Experimental design. **(B)** Tumor progression at phase-1 (*n* = 4–5). **(C)** Tumor progression at phase-2 (*n* = 3–4). **(D)** Detection of memory CD8 T cells (gated as CD8^+^CD44^hi^) in blood of tumor-bearing mice at phase-2. **(E)** Quantification of D (*n* = 3–4). Representative of two experiments (A–E). Data are presented as the mean ± SEM. *, P < 0.05; **, P < 0.01; ***, P < 0.001; ****, P < 0.0001 by one-way ANOVA (B) or Student’s *t* test (E). See also Fig. S5.

Many successful and in-development cancer immunotherapies target metabolic reprogramming of immune response in the tumor microenvironment (McCarthy et al., 2013; Ho and Kaech, 2017; Kishton et al., 2017; Patel and Powell, 2017). In particular, checkpoint blockade therapies, such as PD-1/PD-L1 blockade therapies, have been indicated to correct the glucose usage imbalance between tumor cells and T cells by altering glycolysis and directing the energy metabolism to favor T cells (Gubin et al., 2014; Chang et al., 2015; Baumeister et al., 2016; Bengsch et al., 2016; Scharping et al., 2016). By providing a potent and nonredundant energy buffering benefit for tumor-fighting T cells, we postulate that creatine supplementation may synergize with a PD-1/PD-L1 blockade therapy to further improve cancer treatment efficacy. Indeed, in a mouse MC38 colon cancer model sensitive to PD-1/PD-L1 blockade therapy (Homet Moreno et al., 2016), the combination of creatine supplementation and anti–PD-1 treatment generated a significant tumor suppression effect superior to that of each treatment alone (Fig. 6, A and B). In fact, most (four of five) experimental mice receiving the combination therapy completely eradicated their tumor burden and remained tumor-free for >3 mo (Fig. 6 C). When receiving a second challenge of MC38 tumor cells, all these “cancer survivors” were protected from tumor recurrence and stayed tumor-free for another 6 mo, the duration of the experiment (Fig. 6 C). This appealing tumor protection effect was associated with a significant increase of memory-phenotype CD8 T cells in the surviving mice, most likely generated from the successful antitumor T cell response in the initial tumor challenge and later on used by the surviving mice to fight off a second tumor challenge (Fig. 6, D and E). Collectively, these encouraging results suggest a promising potential of creatine supplementation for combination cancer immunotherapy.

## Discussion

**Figure 7.**
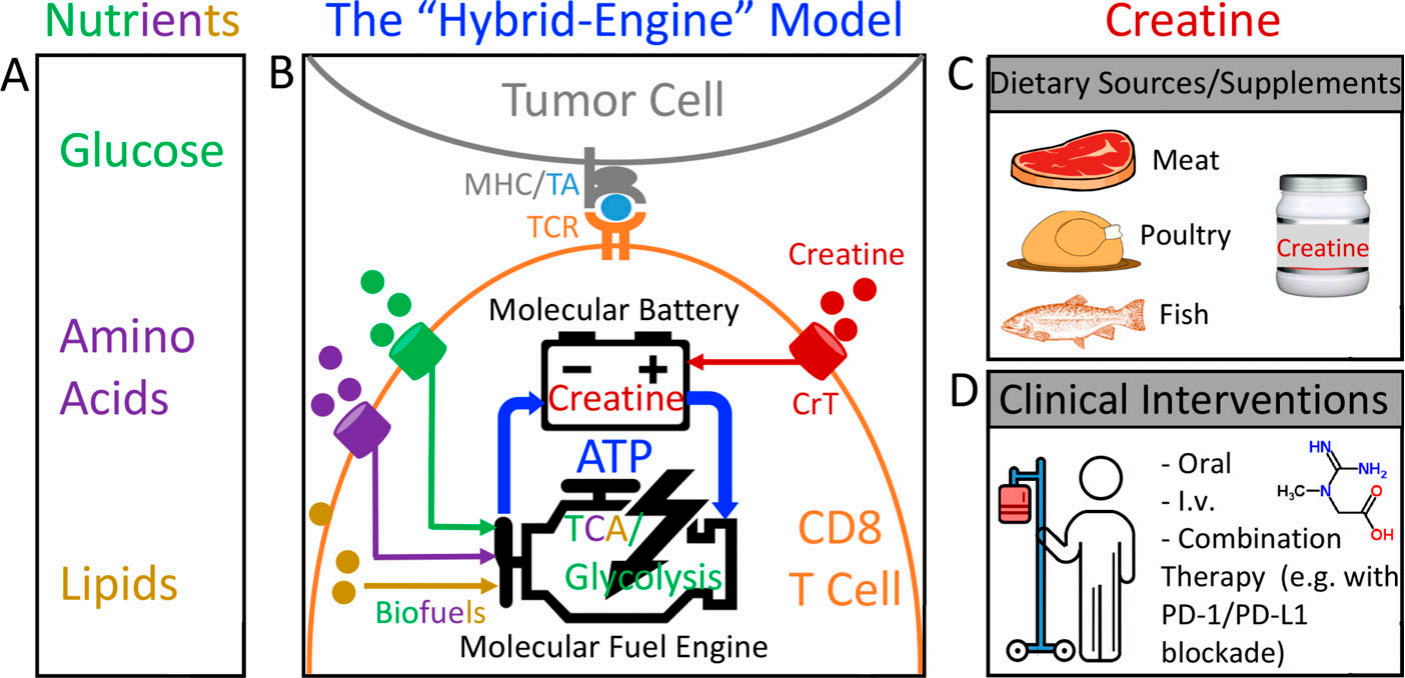
**The hybrid engine model: an updated view of the molecular machinery that powers antitumor T cell immunity. (A)** Nutrients that serve as the biofuels, which can be limiting in the tumor microenvironment. **(B)** The hybrid engine model. To analogize the hybrid car, a tumor-targeting CD8 T cell utilizes a “molecular fuel engine,” such as aerobic glycolysis and/or tricarboxylic acid cycle, to convert nutrients/biofuels into bioenergy in the form of ATP, while using creatine as a “molecular battery” to store bioenergy and buffer the intracellular ATP level to power T cell antitumor activities. **(C)** Creatine can be obtained from creatine-rich dietary resources, mainly red meat, poultry, and fish, as well as from dietary supplements. **(D)** However, the best cancer therapy benefits would come from clinical intervention by administering creatine to cancer patients following specially designed dosing strategies.

Based on our findings, we propose a “hybrid engine” model to update the molecular machinery that powers antitumor T cell immunity by incorporating creatine into the picture (Fig. 7). Analogous to the popular hybrid car, which uses two distinct sources of power, a tumor-targeting CD8 T cell utilizes a “molecular fuel engine” such as glycolysis and/or tricarboxylic acid cycle to convert nutrients/biofuels (e.g., glucose, amino acids, and lipids) into bioenergy in the form of ATP, while using creatine as a “molecular battery” to store bioenergy and buffer the intracellular ATP level, to support T cell antitumor activities (Fig. 7 B). This hybrid engine system is energy efficient, enabling a tumor-targeting CD8 T cell to make maximal use of its available bioenergy supply and perform in a metabolically stressful microenvironment where it has to compete with fast-growing tumor cells for a limited supply of nutrients (Fig. 7 A; Fox et al., 2005; Siska and Rathmell, 2015; Wherry and Kurachi, 2015). CD8 T cells have limited capacity to de novo synthesize creatine; therefore, they heavily rely on uptake of creatine from extracellular resources via CrT (Fig. 7 B), all of which opens up the possibility of reinvigorating tumor-fighting CD8 T cells through creatine supplementation. Creatine can be obtained from creatine-rich dietary resources, mainly red meat, poultry, and fish, as well as from dietary supplements (Wyss and Kaddurah-Daouk, 2000; Kreider et al., 2017; Fig. 7 C). However, the best cancer therapy benefits would come from clinical intervention by administering creatine to cancer patients following specially designed dosing strategies (Fig. 7 D). Both oral and direct administration (e.g., intravenous) routes can be effective (Fig. 7 D).

Our study showed that creatine supplementation suppressed tumor growth in multiple mouse tumor models, including the B16 melanoma model (Fig. 5) and the MC38 colon cancer model (Fig. 6), suggesting that this treatment may provide a general therapeutic benefit to many different types of cancer. Moreover, because creatine works through a novel “energy-buffering” mechanism that is nonredundant to the mechanisms used by many successful and in-development immunotherapies, creatine supplementation can potentially become an effective and economical common component for combination cancer immunotherapies. In our study, we showed that creatine supplementation synergized with checkpoint blockade therapies such as the PD-1/PD-L1 blockade therapy to yield superior therapeutic efficacy (Fig. 6). Many other cancer therapeutic modalities, including the booming new immunotherapies as well as traditional chemotherapies and radiation therapies, may also benefit from combining with creatine supplementation treatment (Fig. 7 D; Pardoll, 2012; Couzin-Frankel, 2013; Page et al., 2014; Ribas, 2015; Rosenberg and Restifo, 2015; Baumeister et al., 2016; Lim and June, 2017).

In the past three decades, oral creatine supplements have been broadly used by bodybuilders and athletes to gain muscle mass and improve performance (Wyss and Kaddurah-Daouk, 2000; Kreider et al., 2017). The new discovery that creatine supplementation may help build a stronger immune system in addition to building a stronger body is exciting. For the active users of creatine supplements, this discovery means possible additional health benefits; for disease patients, it means new immunotherapeutic opportunities. The well-documented safety of long-term creatine supplementation in humans affords a green light for using creatine supplementation to treat chronic diseases such as cancer (Kreider et al., 2017). Meanwhile, the muscle enhancement effect of creatine supplementation, as demonstrated from human experience and shown in our animal studies (Fig. 5, F and G), may also benefit cancer patients who at their late stages often suffer from cachexia, or wasting syndrome (de Campos-Ferraz et al., 2014). Interestingly, some early studies showed that creatine and creatine analogues could directly inhibit cancer growth, presumably through disrupting cancer cell metabolism, suggesting an additional mechanism that creatine may employ to mediate its antitumor effects (Miller et al., 1993; Kristensen et al., 1999). Conversely, CrT has been suggested as a possible biomarker for circulating tumor cells within the blood, posing the concern that creatine supplement may have potential negative effects on CrT-positive tumors (Riesberg et al., 2016). Interestingly, for the two mouse tumor models used in our study, B16 melanoma cells express CrT (as well as creatine kinase brain form) while MC38 colon cancer cells do not (Fig. S5 B). Creatine supplementation exhibited tumor suppression benefits in both tumor models (Fig. 5 C and Fig. 6 B), suggesting that this therapy may have the potential to treat both CrT-positive and CrT-negative tumors.

The energy-buffering function of creatine certainly goes beyond regulating CD8 T cells. In *CrT*-KO mice, we have observed the hyporesponsiveness of multiple immune cells in various mouse tumor models. It is also likely that creatine regulates immune reactions to multiple diseases beyond cancer, such as infections and autoimmune diseases (Riesberg et al., 2016). Studying the roles of creatine in modulating various immune cells under different health and disease conditions will be interesting topics for future research.

## Materials and methods

### Mice

*C57BL/6J* (B6) and *B6.SJL-Ptprc^a^Pepc^b^/BoyJ* (CD45.1, BoyJ) mice were purchased from the Jackson Laboratory, and 6–10-wk-old mice were used for all experiments, unless otherwise indicated.

*B6(Cg)-Slc6a8^tm1.2Clar^/J* mice*,* referred to as *CrT*-KO mice, were purchased from the Jackson Laboratory (Skelton et al., 2011). The experimental colony was produced by breeding female hemizygous mice with male WT littermates. 6–10-wk-old mice were used for all experiments, unless otherwise indicated. C57BL/6-Tg(TcraTcrb)1100Mjb/J (*OT1* Tg) mice were purchased from the Jackson Laboratory and bred with the *CrT-*KO mice to generate *OT1*Tg*^CrT^*^-WT^ and *OT1*Tg*^CrT^*^-KO^ mice. 6–10-wk-old mice were used for all the experiments, unless otherwise indicated. *NOD.Cg-Prkdc^SCID^Il2rg^tm1Wjl^/SzJ (NOD/SCID/IL-2Rγ*^−^*^/^*^−^*, NSG)* mice were purchased from the Jackson Laboratory. 6–10-wk-old females were used for all experiments, unless otherwise indicated.

The animals were housed under specific pathogen–free conditions with 12-h day/night cycles. All animal experiments were approved by the Institutional Animal Care and Use Committee of the University of California, Los Angeles (UCLA).

### Antibodies and flow cytometry

Fluorochrome-conjugated monoclonal antibodies specific for mouse CD45.2 (109820; clone 104), TCRβ (109220; clone H57-597), CD4 (100531; clone RM4-5), IFN-γ (505806; clone XMG1.2), Granzyme B (372208; clone QA16A02), TCR Vα2 (127809; clone B20.1), CD69 (104508; clone H1.2F3), CD25 (102006; clone PC61), CD8 (100732; clone 53-6.7), CD44 (103030; clone IM7), LAG-3 (CD223; 125207; clone C9B7W), and Tim-3 (CD366; 119705; clone RMT3-23) were purchased from BioLegend. Monoclonal antibodies specific for mouse IL-2 (554428; clone JES6-5H4); TCR Vβ5 (1553190; clone MR9-4); and Fc block (anti-mouse CD16/32; 553142; clone 2.4G2) were purchased from BD Biosciences. Monoclonal antibody specific for mouse PD-1 (12-9981-83; clone RMPI-30) was purchased from the eBioscience. Fixable Viability Dye eFluor 506 (65-0866) was purchased from Thermo Fisher Scientific. Cells were stained with Fixable Viability and Fc blocking dye first, followed by surface marker staining. To detect intracellular molecules (Granzyme B and cytokines), cells were subjected to intracellular staining using a Cell Fixation/Permeabilization Kit (554714; BD Biosciences), following the manufacturer’s instructions. To analyze cell viability, cells were stained with Annexin V and 7-AAD using a FITC Annexin V Apoptosis Detection Kit (640922; BioLegend), following the manufacturer’s instructions. Stained cells were analyzed using a MACSQuant Analyzer 10 Flow Cytometer (Miltenyi Biotec). FlowJo software (TreeStar) was used to analyze the data.

Purified anti-mouse CD3 antibody (100314; clone 145-2C11) used for in vitro stimulation of CD8 T cells was purchased from BD Biosciences. Anti-mouse CD3 depleting antibody (BE0002; clone 17A2) and its isotype control antibody (BE0090; clone LTF-2), as well as anti-mouse PD-1 blocking antibody (BE0146; clone RMP1-14) and its isotype control antibody (BE0089; clone 2A3), used for in vivo animal study were purchased from BioXCell.

### Mouse tumor models

B16-OVA murine melanoma cells (obtained from the laboratory of Pin Wang, University of Southern California, Los Angeles, CA; Liu et al., 2014) and MC38 murine colon adenocarcinoma cells (obtained from the laboratory of Antoni Ribas, UCLA, Los Angeles, CA; Homet Moreno et al., 2016) were cultured in high-glucose (4.5 g/liter) DMEM supplemented with 10% FBS and penicillin-streptomycin (Thermo Fisher Scientific) at 37°C and with 5% CO_2_.

To establish solid tumors, mice were s.c. injected above the right flank with 10^6^ B16-OVA or 3 × 10^5^ MC38 cells. Before injection, cells in log phase of growth were harvested and suspended in PBS, and 50 µl of cell suspension were s.c. injected above the flank. Tumor size was periodically measured with a digital Vernier caliper (Thermo Fisher Scientific).

### BM transfer

BM cells were prepared from femurs and tibias by flushing with 25G needles. BM cells from *CrT*-KO mice were administered by retroorbital injection to BoyJ female recipient mice that had received 1,200-rad total-body irradiation. Control BoyJ recipient mice received BM cells from the *CrT*-WT littermates. In both groups, 8 × 10^6^ *CrT*-WT or *CrT*-KO BM cells were injected into recipient mice. BM recipient mice were housed in a sterile environment and maintained on the combined antibiotics sulfmethoxazole and trimethoprim oral suspension (Septra; Hi-Tech Pharmacal) for 12 wk until analysis or use for further experiments. Blood was collected by retroorbital bleeding and analyzed by flow cytometry to confirm the reconstitution. Tumor inoculation started 12 wk after BM transfer.

### Isolation of OT1 Tg T cells and adoptive T cell transfer

The OT1 Tg T cells were purified from the spleen and lymph node cells of either *OT1*Tg*^CrT-^*^WT^ or *OT1*Tg*^CrT-^*^KO^ mice (denoted as OT1*^CrT^*^-WT^ or OT1*^CrT^*^-KO^ cells, respectively) through magnetic-activated cell sorting (MACS) using a mouse CD8 T Cell Isolation Kit (120117044; Miltenyi Biotec) according to the manufacturer’s instructions. The purified OT1*^CrT^*^-WT^ or OT1*^CrT^*^-KO^ cells were then used for in vitro culture or in vivo adoptive T cell transfer studies.

For adoptive T cell transfer, BoyJ female mice (referred to as recipient mice) were injected s.c. above the right flank with 10^6^ B16-OVA cells. 7 d after tumor inoculation, recipient mice received 600-rad total-body irradiation, followed by retroorbital injection of purified OVA-specific OT1 Tg T cells (10^5^ OT1 T cells per mouse).

### TII cell isolation and analysis

Solid tumors were collected from experimental mice at the termination of each tumor experiment. Tumors were cut into small pieces and smashed against a 70-µm cell strainer (07-201-431; Corning) to prepare single cells. Immune cells were enriched through gradient centrifugation with 50% Percoll (P4937; Sigma-Aldrich) at 800 *g* for 30 min at room temperature without brake, followed by treatment with Tris-buffered ammonium chloride buffer to lyse red blood cells according to a standard protocol (Cold Spring Harbor Protocols). The resulting TIIs were then used for further analysis.

To assess gene expression, CD45^+^ immune cells were sorted from TIIs using flow cytometry and then analyzed for *CrT* mRNA expression using qPCR. To assess T cell activation status, TIIs were analyzed for surface activation marker (CD25 and PD-1) expression using flow cytometry. To assess T cell cytotoxicity, TIIs were analyzed for intracellular Granzyme B expression using flow cytometry. To assess T cell cytokine production, TIIs were stimulated with PMA (50 ng/ml) + ionomycin (500 ng/ml) in the presence of GolgiStop (4 µl per 6-ml culture) for 4 h, then analyzed for intracellular cytokine (IL-2 and IFN-γ) production using flow cytometry. CD8 T cells were identified by costaining TIIs with cell surface lineage markers (gated as CD45^+^TCRβ^+^CD4^−^CD8^+^ cells).

### CD8 T cell isolation, in vitro culture, and analysis

Spleen and lymph node cells were harvested from experimental mice and were subjected to MACS using a mouse CD8 T Cell Isolation Kit (Miltenyi Biotec) according to the manufacturer’s instructions. The resulting purified CD8 T cells were then used for in vitro culture and analysis.

CD8 T cells were cultured in vitro in standard T cell culture medium comprising RPMI 1640 (10040; Corning), 10% FBS (F2442; Sigma-Aldrich), 1% penicillin-streptomycin-glutamine (10378016; Gibco), 1% MEM Non-Essential Amino Acids Solution (11140050; Gibco), 1% Hepes (15630080; Gibco), 1% sodium pyruvate (100 mM; 11360070; Gibco), and 0.05 mM β-mercaptoethanol (M3148; Sigma-Aldrich). Unless otherwise indicated, cells were seeded at 0.5 × 10^6^ cells per well in 24-well plates and stimulated with plate-bound anti-CD3 (5 µg/ml; clone 145-2C11), for ≤5 d. At indicated time points, cells were collected and analyzed for *CrT* mRNA expression using qPCR, for cell proliferation through cell counting, for viability through Annexin V/7-AAD staining followed by flow cytometry analysis, for surface activation marker (CD25) expression through surface staining followed by flow cytometry analysis, for effector molecule (Granzyme B, IL-2, and IFN-γ) production through intracellular staining followed by flow cytometry analysis, and for cytokine (IL-2 and IFN-γ) secretion through collecting cell culture supernatants followed by ELISA analysis. CrT protein expression and TCR signaling events were analyzed using Western blot analysis.

In some experiments, ATP (A6419; Sigma-Aldrich) was reconstituted in sterile PBS and added to T cell culture (100 µM) for 2–3 d along with anti-CD3 stimulation, followed by analyzing T cell surface activation marker (CD25) expression using flow cytometry and effector cytokine (IFN-γ) secretion using ELISA. In some experiments, T cells were stimulated with anti-CD3 for 48 h, rested at 4°C for 2 h, then restimulated with anti-CD3 for 30 min in the presence or absence of ATP supplementation (100 µM) followed by analyzing TCR signaling events using Western blot.

In some other experiments, AICAR (A9978; Sigma-Aldrich), an AMPK activator, was reconstituted in DMSO and used to pretreat T cells for 30 min at a concentration of 2 mM, followed by 20 min of anti-CD3 stimulation for Western blot analysis of TCR signaling events, or at a concentration of 250 µM followed by 16 h of anti-CD3 stimulation for flow cytometry analysis of CD25 expression and ELISA analysis of IL-2 production.

For in vitro creatine supplementation experiments, creatine monohydrate (C3630; Sigma-Aldrich) was reconstituted in standard T cell culture medium and added to T cell culture. T cells were stimulated with anti-CD3 for 48 h in the presence or absence of creatine supplementation (0.5 mM), rested at 4°C for 2 h, then restimulated with anti-CD3 for 10 min in the presence or absence of creatine supplementation (0.5 mM) followed by TCR signaling events analysis using Western blot.

### MIG mock and MIG-*CrT* retroviruses

MIG mock retroviral vector was reported previously (Smith et al., 2015; Li et al., 2017). MIG vector is derived from the murine stem cell virus and contains an internal ribosome entry site linked to an enhanced GFP reporter gene. The MIG-*CrT* construct was generated by inserting the mouse *CrT* (*Slc6A8*) cDNA (codon-optimized; synthesized by IDT) into the MIG retroviral vector. The CrT cloning sequences are as follows: forward, 5′-GTC​TCT​CCC​CCT​TGA​ACC​TCC​TCG​TTC-3′, and reverse, 5′-CAA​GCG​GCT​TCG​GCC​AGT​AAC​G-3′. Retroviruses were produced using HEK293T cells following a standard calcium precipitation method (Smith et al., 2015; Li et al., 2017). For viral transduction, CD8 T cells isolated from the spleen and lymph nodes of *CrT*-KO mice were stimulated in vitro with plate-bound anti-CD3 (5 µg/ml) for 4 d. On days 2 and 3 following stimulation, cells were spin infected with retroviral-containing supernatants supplemented with 10 µg/ml polybrene (TR-1003-G; Millipore) for 90 min at 770 *g* at 30°C. On day 4, cells were collected for analysis.

### mRNA qPCR analysis

Total RNA was isolated using TRIzol reagent (15596018; Invitrogen, Thermo Fisher Scientific) according to the manufacturer’s instructions. cDNA was prepared using a SuperScript III First-Strand Synthesis Supermix Kit (18080400; Invitrogen, Thermo Fisher Scientific). Gene expression was measured using a KAPA SYBR FAST qPCR Kit (KM4117; Kapa Biosystems) and a 7500 Real-time PCR System (Applied Biosystems) according to the manufacturers’ instructions. *Ube2d2* (for T cells) or *Actb* (for tumor cells) was used as an internal control. qPCR was performed using the following primers: *CrT* forward, 5′-ACT​GGG​AGG​TGA​CCT​TGT​GC-3′, and reverse, 5′-CGA​TCT​TTC​CTG​TTG​ACT​TG-3′; *Ckb* forward, 5′-AGT​TCC​CTG​ATC​TGA​GCA​GC-3′, and reverse, 5′-GAA​TGG​CGT​CGT​CCA​AAG​TAA-3′, *Agat* forward, 5′-GCT​TCC​TCC​CGA​AAT​TCC​TGT-3′, and reverse, 5′-CCT​CTA​AAG​GGT​CCC​ATT​CGT-3′; *Gamt* forward, 5′-CAC​GCA​CCT​GCA​AAT​CCT​G-3′, and reverse, 5′-CAC​GCA​CCT​GCA​AAT​CCT​G-3′; *Ube2d2* forward, 5′-ACA​AGG​AAT​TGA​ATG​ACC​TGG​C-3′, and reverse, 5′-CAC​CCT​GAT​AGG​GGC​TGT​C-3′; and *Actb* forward, 5′-AGG​TGT​GCA​CCT​TTT​ATT​GGT-3′, and reverse, 5′-TGT​ATG​AAG​GTT​TGG​TCT​CCC-3′. The relative expression of the mRNA of interest was calculated using the 2^ΔΔCT^ method.

### ELISA

ELISA was performed for the detection of cytokines according to a BD Biosciences protocol. The coating and biotinylated antibodies for the detection of mouse IFN-γ (coating antibody, 554424; biotinylated detection antibody, 554426) and IL-2 (coating antibody, 551216; biotinylated detection antibody, 554410) were purchased from BD Biosciences. The streptavidin-HRP conjugate (18410051) was purchased from Invitrogen. Mouse IFN-γ and IL-2 standards were purchased from eBioscience. The 3,3′,5,5′-tetramethylbenzidine (51200048) substrate was purchased from KPL. The absorbance was measured at 450 nm using an Infinite M1000 microplate reader (Tecan).

### Western blot

Total protein was extracted using radioimmunoprecipitation assay lysis buffer (Thermo Fisher Scientific) supplemented with a phosphatase inhibitor cocktail (Sigma-Aldrich) and a protease inhibitor cocktail (Roche) following the manufacturers’ instructions. Nuclear protein was extracted using a Nuclear Protein Extraction Kit (Thermo Fisher Scientific) following the manufacturer’s instructions, or using homemade reagents (10 mM Hepes, pH 7.9, 10 mM KCl, 0.34 M sucrose, 10% glycerol, 1 mM dithiothreitol, 0.1% Triton X-100, 1.5 mM MgCl_2_, and protease inhibitor cocktail) following a previously established protocol (Ma et al., 2019). Protein concentration was measured by a BCA assay (23228 and 1859078; Thermo Fisher Scientific). Equal amounts of protein were resolved on a 12% SDS-PAGE gel and then transferred to a polyvinylidene difluoride membrane by electrophoresis. The following anti-mouse antibodies were purchased from Cell Signaling Technology and used to blot for the protein of interest: p-Zap-70 (2705S; clone 99F2); Zap-70 (2717S; clone Y319); p-Lck (2751S; clone Y505); Lck (2752S); p-c-Jun (9261S; clone S63); NFAT (4389S); NF-κB p65 (8242P; clone D14E12); AMPK (5831T; clone D5A2); p-AMPK (2535T; clone 40H9), secondary anti-mouse (7076P2), and secondary anti-rabbit (7074P2). Anti-mouse CrT (SLC6A8; PA5-37060) was purchased from Thermo Fisher Scientific. β-Actin (sc-69879; clone AC-15; Santa Cruz Biotechnology) was used as an internal control for total protein extracts, while Lamin A (sc-71481; clone 4A58; Santa Cruz Biotechnology) was used as an internal control for nuclear protein extracts. Signals were visualized with autoradiography using an enhanced chemiluminescence system (RPN2232; Thermo Fisher Scientific). Data analysis was performed using ImageJ software (National Institutes of Health).

### ATP quantification

A Luminescent ATP Detection Assay Kit (ab113849; Abcam) was used to quantify intracellular ATP, following the manufacturer’s instructions. The total amount of ATP detected was then normalized to cell numbers.

### Creatine quantification

A Creatine Assay Kit (ab65339; Abcam) was used to quantify creatine, both in vivo and in vitro*,* following the manufacturer’s instructions. For the in vivo study, whole blood was collected (retroorbital bleeding) from the experimental mice in a capillary tube, and the isolated serum was immediately used for the assay following the manufacturer’s directions. For the in vitro study, cells were spun to remove culture media and suspended in cold PBS. Creatine was then quantified following the manufacturer’s directions. The total amount of creatine detected was then normalized to cell numbers.

### In vivo study of creatine supplementation for cancer immunotherapy

For creatine supplementation via i.p. injection, creatine monohydrate (C3630; Sigma-Aldrich) was dissolved in sterile PBS and injected i.p. in experimental animals daily at a dose of 10.5 mg per animal per injection. For creatine supplementation via diet, experimental animals were fed a creatine-enriched isocaloric diet, which is a customized formulation based on PicoLab Rodent Diet 20 enriched in creatine (3 g/kg diet, TD.170082; Envigo Teklad Diet). The diet was designed to reflect the safe daily dose of creatine recommended for enhanced athletic performance in humans (Mayo Clinic data). Nontreated mice (control) were fed a control diet prepared in a manner similar to that of the creatine-enriched diet.

To study the effects of creatine supplementation on suppressing tumor growth, B6 mice were inoculated with B16-OVA tumor cells and monitored for tumor growth, with or without receiving creatine supplementation via i.p. injection or diet. To study the requirement of an immune system for creatine supplementation–induced antitumor effects, B16-OVA tumor growth was compared between B6 mice and immune-compromised NSG mice receiving i.p. supplementation of creatine. To study the T cell dependence of creatine supplementation–induced antitumor effects, B6-OVA tumor growth was monitored and compared in B6 mice receiving i.p. injection of an anti-CD3 T cell–depleting antibody (clone RMP1-14; 100 µg/mouse/injection, twice per wk) or an isotype control antibody (clone LTF-2, 100 µg/mouse/injection, twice per wk), with or without i.p. supplementation of creatine.

To study the combination effects of creatine supplementation and PD-1/PD-L1 blockade treatment, B6 mice were inoculated with MC38 tumor cells and monitored for tumor growth; experimental mice also received i.p. supplementation of creatine, as well as i.p. injection of an anti–PD-1 blocking antibody (clone RMP1-14; 300 µg/mouse/injection, twice per wk) or an isotype control antibody (clone 2A3; 300 µg/mouse/injection, twice per wk), alone or in combination. Tumor-free mice were maintained for 3 mo, then challenged with MC38 tumor cells again and monitored for tumor recurrence over another 6-mo period.

### Histological analysis

Skeletal muscle (biceps femoris) harvested from control and experimental (creatine i.p. and food) mice were fixed in 10% neutral-buffered formalin and embedded in paraffin for sectioning (5-µm thickness), followed by H&E staining using standard procedures (UCLA Translational Pathology Core Laboratory). The sections were imaged using an Olympus BX51 upright microscope equipped with a Macrofire charge-coupled device camera (Optronics). The muscle-fiber diameter was assessed with the use of ImageJ.

### Quantification and statistical analysis

FlowJo software (TreeStar) was used for the analysis of FACS data. ImageJ was used to quantify Western blots and muscle H&E sections. GraphPad Prism 6 was used for graphic representation and statistical analysis of the data. Pairwise comparisons were made using a two-tailed Student’s *t* test. Multiple comparisons were performed using an ordinary one-way ANOVA, followed by Tukey’s multiple comparisons test. Data are presented as the mean ± SEM, unless otherwise indicated. A P value <0.05 was considered significant. *, P < 0.05; **, P < 0.01; ***, P < 0.001; ****, P < 0.0001.

### Online supplemental material

Fig. S1 shows the characterization of *CrT*-KO mice, the study of tumor growth in *CrT*-WT and *CrT*-KO mice without creatine supplementation, and the *CrT* mRNA expression in tumor-infiltrating CD8 T cell subsets. Fig. S2 shows the BM transfer experiment studying whether CrT deficiency in the immune system directly impacts tumor growth. The figure also shows additional data studying the in vivo antitumor capacity of *CrT*-WT and *CrT*-KO OT1 Tg T cells. Fig. S3 shows the creatine level in standard T cell culture medium and the in vitro activation of *CrT*-WT and *CrT*-KO antigen-specific CD8 T cells. The figure also shows additional data studying *CrT*-KO CD8 T cells transduced with MIG-*CrT* retrovector. Fig. S4 shows the study of *CrT*-WT CD8 T cell activation with ATP supplementation, the study of *CrT*-WT and *CrT*-KO CD8 T cell activation with or without AICAR treatment, and the study of *CrT*-WT and *CrT*-KO CD8 T cell proximal signaling activation with or without creatine treatment. Fig. S5 shows the in vivo depletion of T cells in B6 mice using an anti-CD3 depleting antibody, and the study of *CrT* and *Ckb* mRNA expression in B16-OVA and MC38 tumor cells.

## Supplementary Material

Supplemental Materials (PDF)
