# Supplementary material for: Creatine uptake regulates CD8 T cell antitumor immunity
Source: J Exp Med. 2019 Oct 18;216(12):2869–82. doi: 10.1084/jem.20182044 (PMC6888972; doi:10.1084/jem.20182044)
Supplement: Supplemental Materials (PDF) [file JEM_20182044_sm.pdf]

JEM

Supplemental material

Di Biase et al., https://doi.org/10.1084/jem.20182044

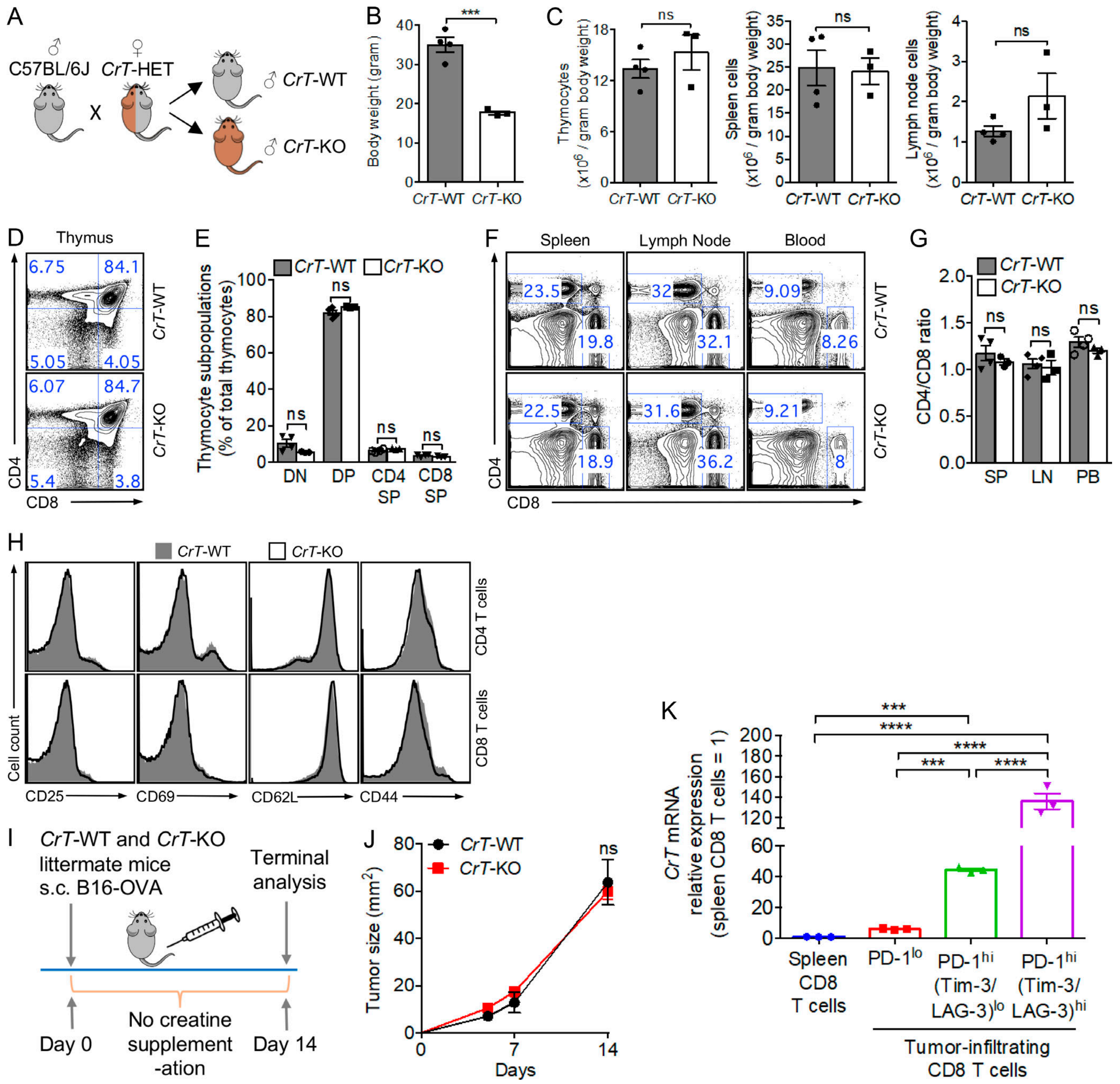

Figure S1. ***CrT*-KO mice show impeded control of tumor growth.** Related to Fig. 1. **(A–H)** Characterization of *CrT*-KO mice. **(A)** Breeding strategy for the generation of *CrT*-KO mice. **(B–H)** Characterization of *CrT*-KO mice in comparison with their *CrT*-WT littermate controls (n = 3–4). **(B)** *CrT*-KO mice showed reduced body weight. **(C)** *CrT*-KO mice contained normal numbers of immune cells proportional to their body weight. **(D and E)** *CrT*-KO mice showed normal T cell development in thymus. **(D)** FACS plot showing the developmental stages of thymocytes defined by CD4/CD8 coreceptor expression. **(E)** Quantification of D. **(F and G)** *CrT*-KO mice contained normal levels of CD4 and CD8 T cells in the periphery. **(F)** FACS plots showing the detection of CD4 and CD8 T cells in the periphery. **(G)** Quantification of F. **(H)** Peripheral T cells in the *CrT*-KO mice displayed a normal naive T cell phenotype ($CD25^{lo}CD69^{lo}CD62L^{hi}CD44^{lo}$). FACS plots of peripheral blood T cells are shown. **(I and J)** Study of B16-OVA tumor growth in *CrT*-WT and *CrT*-KO littermate mice without creatine supplementation. **(I)** Experimental design. **(J)** Tumor growth (n = 3). **(K)** Study of *CrT* gene expression in tumor-infiltrating CD8 T cell subsets. B6 mice were inoculated with B16-OVA tumor cells. On day 19, TIIs were isolated, and CD8 T cells (pregated as $CD45.2^{+}TCR\beta^{+}CD8^{+}$ cells) were sorted into three subsets: PD-1^{lo}, PD-1^{hi} (Tim-3/LAG-3)^{lo}, and PD-1^{hi} (Tim-3/LAG-3)^{hi}. CD8 T cells (gated as $CD45.2^{+}TCR\beta^{+}CD8^{+}$ cells) sorted from the spleen of age-matched, tumor-free B6 mice were included as a control. qPCR analysis of *CrT* mRNA expression in the indicated CD8 T cells is presented (n = 3). Representative of two experiments (A–K). Data are presented as the mean ± SEM. ns, not significant; ***, P < 0.001; ****, P < 0.0001 by Student's t test (B, C, E, G, and J), or one-way ANOVA (K).

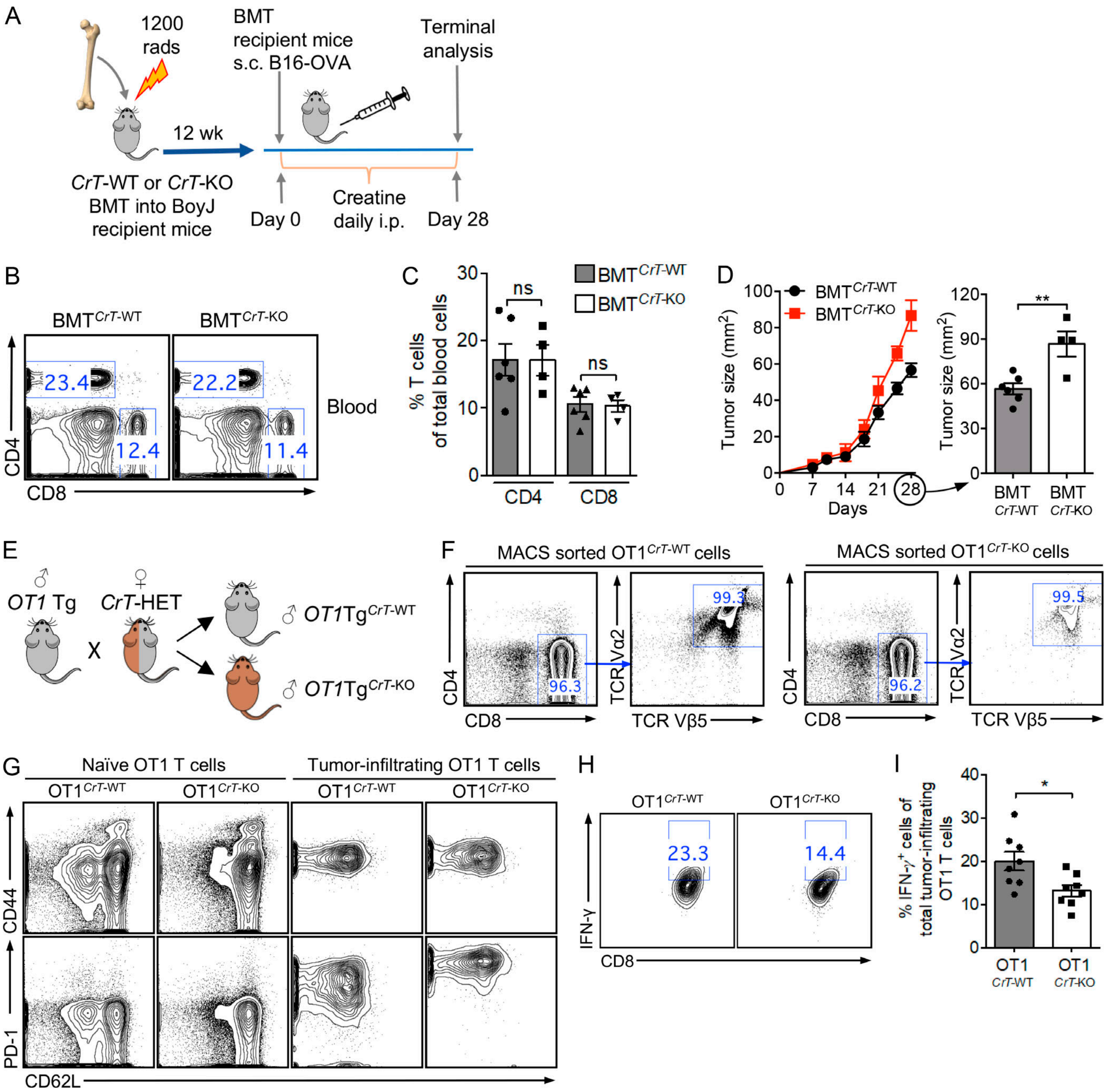

Figure S2. **Creatine uptake deficiency directly impairs antitumor T cell immunity.** Related to Fig. 2. **(A–D)** Studying B16-OVA tumor growth in BoyJ mice receiving adoptive transfer of BM cells from *CrT*-WT or *CrT*-KO donor mice (BMT$^{CrT\text{-WT}}$ or BMT$^{CrT\text{-KO}}$ mice). **(A)** Experimental design. **(B and C)** Characterization of BMT$^{CrT\text{-WT}}$ and BMT$^{CrT\text{-KO}}$ mice. *CrT* deficiency did not impair the capacity of BM cells to reconstitute the T cell compartment in BoyJ recipient mice. **(B)** FACS plots showing the detection of CD4 and CD8 T cells in blood (gated as CD4$^+$ and CD8$^+$ cells, respectively). **(C)** Quantification of B (*n* = 4–6). **(D)** Tumor growth (*n* = 4–6). **(E–I)** Studying the antitumor capacity of *CrT*-WT and *CrT*-KO *OT1* Tg T cells (related to Fig. 2, A–H). **(E)** Breeding strategy for the generation of *OT1* Tg (*OT1* Tg) mice deficient in *CrT* gene (*OT1*Tg$^{CrT\text{-KO}}$ mice), in contrast to the conventional *OT1* Tg mice (*OT1*Tg$^{CrT\text{-WT}}$ mice). **(F)** FACS plots showing the isolation of *OT1* Tg T cells (>99% purity, gated as CD4$^-$CD8$^+$TCR Vα2$^+$TCR Vβ5$^+$ cells) from *OT1*Tg$^{CrT\text{-WT}}$ mice (OT1$^{CrT\text{-WT}}$ cells) and *OT1*Tg$^{CrT\text{-KO}}$ mice (OT1$^{CrT\text{-KO}}$ cells) using MACS. **(G–I)** On day 20 after tumor inoculation, B16-OVA tumors were collected from experimental mice, and TIIs were isolated for further analysis. OT1 Tg T cells were identified as CD45.2$^+$CD8$^+$ cells. **(G)** Studying the impact of *CrT* deficiency on OT1 T cell infiltration into tumor. Representative FACS plots were presented. Compared with OT1$^{CrT\text{-WT}}$ cells, OT1$^{CrT\text{-KO}}$ cells displayed a similar antigen-experienced phenotype (CD44^{hi}CD62L^{lo}) but exhibited a more exhaustion-prone characteristic, as shown by the higher expression of PD-1. **(H and I)** Studying the impact of *CrT* deficiency on functionality of tumor-infiltrating OT1 T cells. **(H)** FACS plots showing the measurements of intracellular IFN-γ. Before intracellular cytokine staining, TIIs were stimulated with PMA and ionomycin in the presence of GolgiStop for 4 h. **(I)** Quantification of H (*n* = 8). Representative of two experiments (A–I). Data are presented as the mean ± SEM. ns, not significant; *, $P < 0.05$; **, $P < 0.01$ by Student's *t* test.

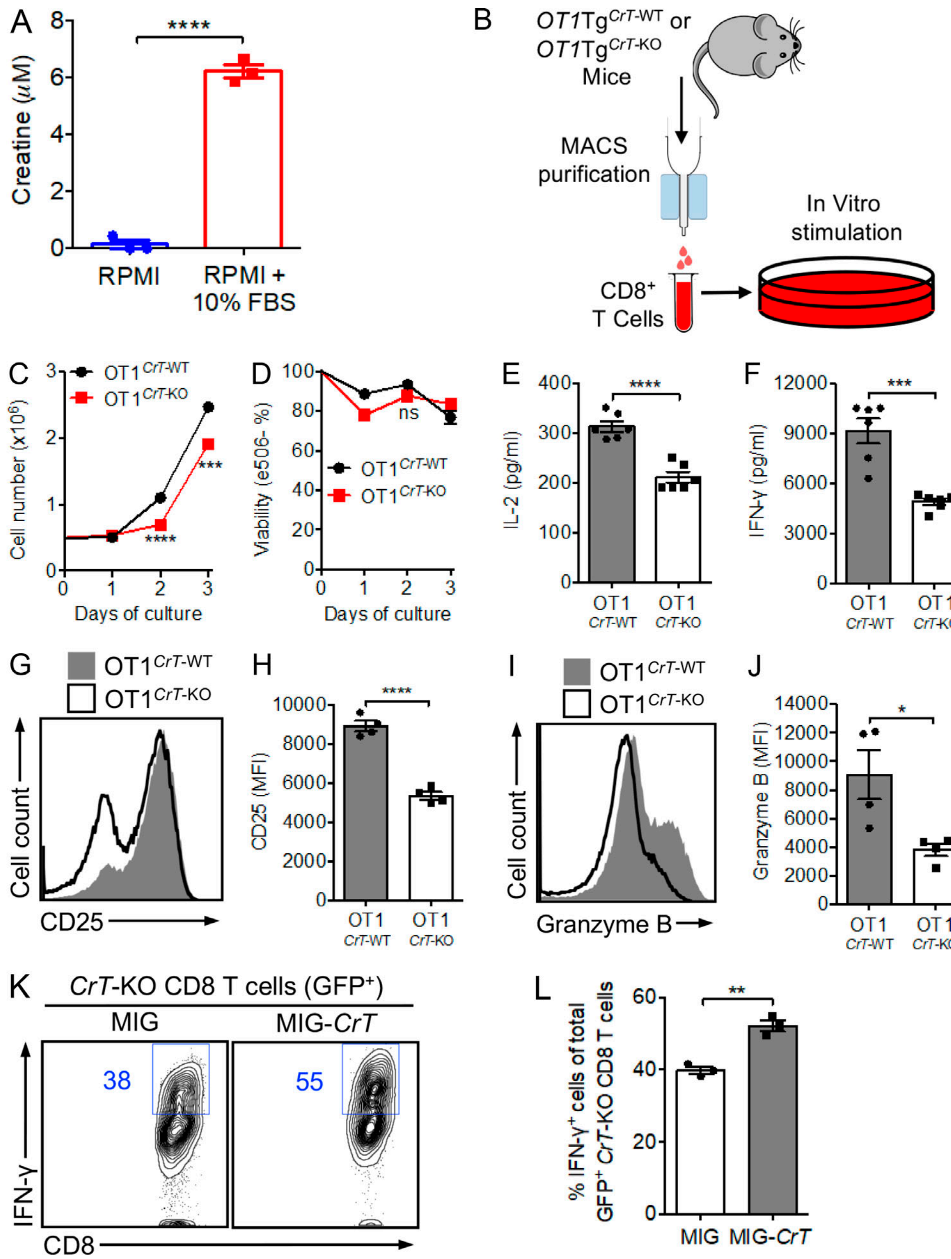

Figure S3. **Creatine uptake regulates CD8 T Cell response to antigen stimulation.** Related to Fig. 3. **(A)** Creatine levels in standard T cell culture medium, measured using a Creatine Assay Kit (Abcam). Note FBS was the source of creatine. **(B–J)** Study of creatine uptake regulation of antigen-specific CD8 T cell response. OVA-specific *OT1* Tg CD8 T cells were isolated from the *OT1*Tg$^{CrT\text{-}WT}$ or *OT1*Tg$^{CrT\text{-}KO}$ mice (denoted as OT1$^{CrT\text{-}WT}$ or OT1$^{CrT\text{-}KO}$ cells, respectively) and then stimulated in vitro with anti-CD3. **(B)** Schematic of the experimental design to isolate OT1$^{CrT\text{-}WT}$ and OT1$^{CrT\text{-}KO}$ cells for in vitro stimulation. **(C–J)** The analyses of cell proliferation (C; *n* = 3), cell viability (D; *n* = 4), effector cytokine production (E and F; *n* = 6), surface CD25 activation marker expression (G and H; *n* = 4), and cytotoxic molecule production (I and J; *n* = 4) are shown. Data of E–J were collected at 48 h after stimulation. MFI, mean fluorescence intensity. **(K and L)** Study of *CrT*-KO CD8 T cells transduced with MIG-*CrT* retrovector (related to main Fig. 3, O–S). **(K)** FACS plots showing the intracellular staining of IFN-γ effector cytokine in GFP+ *CrT*-KO CD8 T cells 96 h after anti-CD3 stimulation and MIG-*CrT* transduction. **(L)** Quantification of K (*n* = 3). Representative of two experiments (A–L). Data are presented as the mean ± SEM. ns, not significant; *, P < 0.05; **, P < 0.01; ***, P < 0.001; ****, P < 0.0001 by Student's *t* test.

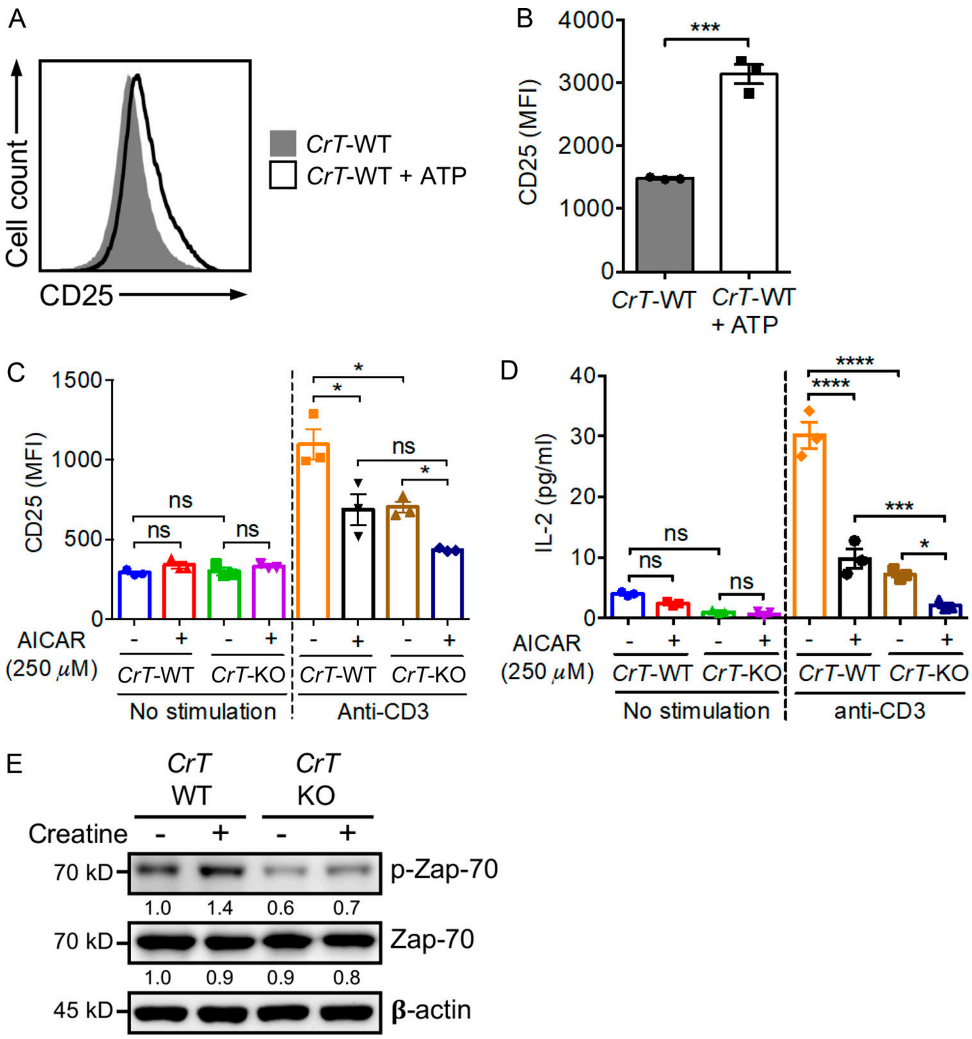

Figure S4. **Creatine uptake regulates CD8 T cell response by regulating T cell ATP/energy buffering.** Related to Fig. 4. **(A and B)** Study of *CrT*-WT CD8 T cell activation with ATP supplementation. *CrT*-WT CD8 T cells were stimulated with anti-CD3, with or without ATP supplementation (100 μM) in the culture medium, and analyzed for surface CD25 activation marker at 48 h. **(A)** FACS plots showing CD25 expression. **(B)** Quantification of A (*n* = 3). Note that ATP supplementation further increased the activation of *CrT*-WT CD8 T cells. MFI, mean fluorescence intensity. **(C and D)** Study of *CrT*-WT and *CrT*-KO CD8 T cell activation with or without AICAR treatment. *CrT*-WT and *CrT*-KO CD8 T cells were pretreated with DMSO or AICAR (250 μM) for 30 min followed by stimulation with anti-CD3 for 16 h. DMSO, solvent used to dissolve AICAR. **(C)** CD25 activation marker expression measured using flow cytometry (*n* = 3). **(D)** IL-2 production measured using ELISA (*n* = 3). (**E**) Study of TCR proximal signaling events in *CrT*-WT and *CrT*-KO CD8 T cells with or without creatine supplementation. Purified *CrT*-WT and *CrT*-KO CD8 T cells were stimulated in vitro with anti-CD3 in the presence or absence of creatine supplementation (0.5 mM) for 48 h, rested at 4°C for 2 h, then restimulated with anti-CD3 in the presence or absence of creatine (0.5 mM) for 10 min. Representative Western blot images showing the analysis of Zap-70 phosphorylation were presented. Representative of two experiments (A–E). Data are presented as the mean ± SEM. ns, not significant; *, P < 0.05; ***, P < 0.001; ****, P < 0.0001 by Student's *t* test (B) or one-way ANOVA (C and D).

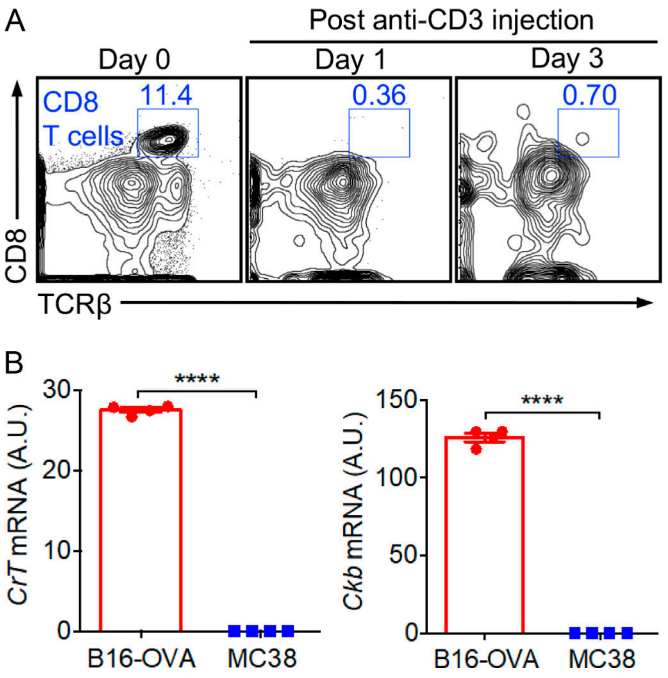

Figure S5. **Creatine supplementation for cancer immunotherapy.** Related to Figs. 5 and 6. **(A)** Studying the requirement of T cells for cancer therapy effects (related to Fig. 5, J and K). Anti-CD3 monoclonal antibody (αCD3, clone 17A2) was used for in vivo depletion of T cells. FACS plots are presented showing the depletion of T cells, in particular CD8 T cells (gated as $TCR\beta^{+}CD8^{+}$), in peripheral blood of experimental mice after receiving i.p. injection of anti-CD3. **(B)** Studying *CrT* and *Ckb* mRNA expression in B16-OVA and MC38 tumor cells using qPCR. $n = 4$. A.U., artificial unit relative to *Actb*. Representative of two experiments (A and B). Data are presented as the mean ± SEM. ****, $P < 0.0001$ by Student's t test.
